# Understanding the Role of Activation Loop Mutants in Drug Efficacy for FLT3-ITD

**DOI:** 10.3390/cancers15225426

**Published:** 2023-11-15

**Authors:** Julhash U. Kazi, Lina Al Ashiri, Rituraj Purohit, Lars Rönnstrand

**Affiliations:** 1 Division of Translational Cancer Research, Department of Laboratory Medicine, Lund University, 22381 Lund, Sweden; 2 Lund Stem Cell Center, Department of Laboratory Medicine, Lund University, 22184 Lund, Sweden; 3 Lund University Cancer Centre (LUCC), Lund University, 22381 Lund, Sweden; 4 CSIR-Institute of Himalayan Bioresource Technology, Palampur 176061, India; rituraj@ihbt.res.in; 5 Department of Hematology, Oncology and Radiation Physics, Skåne University Hospital, 22185 Lund, Sweden

**Keywords:** drug sensitivity scores, molecular modeling, molecular docking, four-parameter logistic curve

## Abstract

**Simple Summary:**

In this study, we investigated FLT3, a protein that plays a vital role in the development of early blood cells. FLT3 often undergoes changes that contribute to the onset of a blood cancer known as acute myeloid leukemia (AML). We employed sophisticated computational techniques to examine how various mutations in the FLT3 protein affect its function and its interaction with therapeutic drugs. Our analysis covered numerous combinations of potential FLT3 mutations and drugs to identify the most effective treatments. Specifically, we concentrated on the impact of a mutation at the Y842 site within FLT3 on the efficacy of drug treatments. Furthermore, we introduced a novel scoring system designed to enhance the prediction of drug effectiveness. Our findings highlight the significance of customized medical approaches, considering that individual mutations can markedly alter a patient’s reaction to AML treatments.

**Abstract:**

The type III receptor tyrosine kinase FLT3 is a pivotal kinase for hematopoietic progenitor cell regulation, with significant implications in acute myeloid leukemia (AML) through mutations like internal tandem duplication (ITD). This study delves into the structural intricacies of FLT3, the roles of activation loop mutants, and their interaction with tyrosine kinase inhibitors. Coupled with this, the research leverages molecular contrastive learning and protein language modeling to examine interactions between small molecule inhibitors and FLT3 activation loop mutants. Utilizing the ConPLex platform, over 5.7 million unique FLT3 activation loop mutants—small molecule pairs were analyzed. The binding free energies of three inhibitors were assessed, and cellular apoptotic responses were evaluated under drug treatments. Notably, the introduction of the Xepto50 scoring system provides a nuanced metric for drug efficacy. The findings underscore the modulation of molecular interactions and cellular responses by Y842 mutations in FLT3-KD, highlighting the need for tailored therapeutic approaches in FLT3-ITD-related malignancies.

## 1. Introduction

FLT3, also known as Fms-like tyrosine kinase 3, is a receptor tyrosine kinase that plays a pivotal role in the hematopoietic system. It is predominantly expressed in hematopoietic progenitor cells, acting as a key regulator of their survival, proliferation, and differentiation. This significance of FLT3 has been extensively documented and is reviewed by us and others in several studies [1,2,3,4,5,6,7,8,9]. In the context of acute myeloid leukemia (AML), FLT3 takes on an even more pronounced role. AML, a malignancy of the myeloid lineage of blood cells, exhibits a variety of genetic anomalies. Notably, about 30% of AML patients carry an activating mutation in the FLT3 gene. This mutation significantly boosts the cell’s survival and proliferation capabilities, often leading to aggressive disease progression. The most frequently observed of these mutations is the internal tandem duplication (ITD). This intriguing mutation involves an in-frame duplication of a sequence within the FLT3 gene. This duplication can vary in length, from just a few amino acids to more than a hundred. The result of this mutation is a structural alteration where the juxtamembrane region becomes separated from the kinase domain. Consequently, this change activates the kinase activity, driving the oncogenic properties of the cell. Clinical observations have revealed a grim picture: the presence of an ITD mutation in FLT3 often correlates with poor survival rates and a challenging overall prognosis for AML patients. Diving deeper into the structure of FLT3, within its kinase domain, there lies a conserved tyrosine residue located in what is referred to as the activation loop. This loop is a hallmark of kinase enzymes and is frequently involved in modulating their activity. A wealth of research, including the study referenced as [10], highlights the importance of activation loops across various kinases. However, when it comes to type III receptor tyrosine kinases, a group to which FLT3 belongs, this loop does not play the conventional regulatory role. Our previous research efforts have unveiled that this particular tyrosine residue in FLT3, while not crucial for its kinase activity, is indispensable for the transformative capabilities of the FLT3-ITD mutation [11]. Further complicating the clinical landscape, mutations in codon 842, specifically Y842H and Y842C, have emerged as culprits in mediating resistance to tyrosine kinase inhibitors, a common therapeutic strategy for AML [12]. Among these, the Y842C mutation deserves special mention. It has not only been identified as a mechanism of drug resistance but has also been flagged as an activating mutation in AML patients, as detailed in the study referenced as [13].

The extracellular domain of type III Receptor Tyrosine Kinases (RTKs) is architecturally composed of five immunoglobulin-like (Ig-like) domains. Among these, the Ig-like motifs 2 and 3 are specifically involved in ligand binding, providing specificity to the ligand–receptor interaction. In contrast, domains 4 and 5 have the crucial function of mediating receptor dimerization, a fundamental step for the signaling capabilities of these receptors. Anchoring these receptors firmly to the cell membrane is a hydrophobic transmembrane domain. This domain acts as a gateway between the extracellular environment and the cell’s interior. Adjacent to the transmembrane domain lies the intricate intracellular region. This region starts with the juxtamembrane region and subsequently houses the bipartite kinase domain, ultimately ending with the carboxyterminal tail. For type III RTKs, the juxtamembrane region is not just a mere structural component. It performs a crucial autoinhibitory function. By strategically binding to the activation loop of the kinase domain, it effectively locks the kinase in an inactive state, ensuring that signaling is tightly regulated [14]. When FLT3 is in this inactive state, it remains unphosphorylated. The activation loop adopts a distinct conformation, often referred to as the ‘DFG-out’ conformation due to its conserved aspartic acid-phenylalanine-glycine (DFG) sequence. This loop, approximately 27 residues in length, interacts with the alanine-proline-glutamic acid (APE) sequence, a detail that has been elaborated upon in various reviews, including [15]. In a scenario where FLT3 remains unbound to its ligand and thus inactive, the juxtamembrane region interacts with the kinase domain. This interaction maintains the kinase domain in its inhibited state. Interestingly, this DFG-out conformation has been exploited therapeutically. Tyrosine kinase inhibitors that bind to this conformation are termed type II inhibitors. Imatinib, a prototypical tyrosine kinase inhibitor (TKI), is a classic example of this category. Conversely, there are Type I TKIs that differ in their mechanism. Instead of the DFG-out conformation, they interact with the kinase domain when it is in the “DFG-in” configuration, signifying an active state of the kinase. Within the scope of the TKIs discussed here, Midostaurin is categorized as a type I inhibitor. In contrast, both Sorafenib and Quizartinib fall under the type II inhibitors, emphasizing their distinct binding and inhibitory profiles.

TKIs have shown promise in the clinical treatment of various cancers. However, mutations in the target proteins can compromise the effectiveness of these drugs [1,11,16]. It is essential to grasp the detailed interactions between drugs and target proteins and to pinpoint inhibitors that can selectively target these mutations to mitigate such issues. While conventional drug discovery processes can be time-consuming when searching for mutation-specific drugs, recent advances in computational methods offer the potential for a more expedited approach. One such advanced computational method is molecular contrastive learning (MCL), which has emerged to meet the unique challenges of drug discovery. MCL operates on a self-supervised learning model that enhances the process of representation learning by contrasting aligned pairs (positive) against disparate ones (negative) [17]. A positive pair includes two variations of the same molecular structure, while a negative pair involves variations from different structures. The goal of MCL is to refine the representation so that similar items (positive pairs) are closer in the learned space, while dissimilar items (negative pairs) are further apart. This process effectively prioritizes the association of items with similar meanings while distancing those with different meanings. The strength of MCL lies in its ability to generate nuanced and informative representations of proteins and ligands. These representations can then be used to calculate similarity scores, acting as predictors for potential interactions between proteins and ligands [18]. By facilitating the discovery of new drug–target interactions and aiding in the repurposing of existing drugs, MCL significantly contributes to the field, potentially accelerating the identification of drugs suited to target mutation-specific conditions.

In the quest for mutation-specific drugs and the optimization of TKIs for cancer therapies, molecular dynamics (MD) and molecular docking stand out for their profound impact on computational drug discovery [19]. MD simulations unravel the complex dance of atoms and molecules within drug-target complexes over time, revealing the dynamic nature of their interactions. This dynamic perspective is indispensable when considering the effect of mutations on the target proteins, as it allows for a nuanced exploration of how these genetic alterations might influence drug binding and efficacy. MD sheds light on the nuanced dance of proteins’ conformational changes, assesses the stability of drug molecules within binding pockets, and unveils the subtleties of how mutations can affect both drug accessibility and the strength of binding [20]. Complementing the temporal resolution of MD, molecular docking offers a spatial dimension, predicting how a drug molecule might orient itself to a target protein to form a stable complex. It allows for the precise modeling of interactions between small molecules and proteins down to the level of individual atoms, which is pivotal for a detailed understanding of drug actions and their potential efficacies [21]. In scenarios where mutations are present, docking becomes an invaluable tool for sifting through libraries of compounds to pinpoint those that bind most effectively to altered binding sites, hinting at their selectivity and potency as inhibitors. When integrated, these computational strategies—MD for capturing the dynamic interplay over time and docking for visualizing the static potential of interactions—provide a dual lens through which the interactions of drugs with mutated proteins can be viewed in high definition [22,23]. This synergy not only enriches our understanding of the molecular basis for drug efficacy but also streamlines the drug development pipeline. By predicting which compounds are likely to exhibit strong affinities for particular targets, especially those with specific mutations, researchers can more efficiently prioritize candidates for further development. This harmonized approach propels the drug discovery process forward, enhancing the selection of potential therapeutic agents while conserving valuable time and resources.

In this study, we utilized the MCL-dependent protein language model, ConPLex [24], to identify potential drug candidates targeting FLT3 mutations, specifically at the Y842 position. We employed MD and molecular docking to understand how mutations affect inhibitor interactions. Finally, we conducted viability and apoptosis assays to validate our computational findings and developed a tool named Xepto50 to elucidate the wet lab results (briefly depicted in Figure S1).

## 2. Materials and Methods

### 2.1. Preparation of Native and Mutant FLT3 Structures

The native structure of the FLT3 protein, with a resolution of 3.20 Å, determined through X-ray diffraction was obtained from the Protein Data Bank (PDB) [25]. The PDB ID for the dimeric FLT3 structure is 4XUF [26]. In the structural configuration of FLT3, the fundamental kinase fold comprises a compact N-terminal lobe (N lobe) and an α-helical C-terminal lobe (C lobe) connected through a hinge segment. The pivotal conserved structural components crucial for kinase catalytic activity, situated between the N lobe and the C lobe, encompass the hinge region. For our computational analysis, we utilized only one subunit, specifically Subunit A. The crystallographic structure displayed two missing loops: one between residues Lys649 and Asp651, and the other between Glu708 and Val782. These missing loops were reconstructed using the Modeler plugin within the Chimera software (version chimera-1.3-tru64). The co-crystal ligand, quizartinib, was excised from the binding site. Point mutations were then introduced into the native FLT3 protein structure at position Y842 to produce the Y-to-C and Y-to-F mutant proteins. These mutant structures were generated using the Dunbrack rotamer library [27], and, among them, structures with the lowest energy and highest probability scores were chosen for subsequent computational analyses. The molecular structures of Quizartinib (PubChem CID: 24889392), Sorafenib (PubChem CID: 216239), and Midostaurin (PubChem CID: 9829523) were sourced from the PubChem database [28].

### 2.2. Molecular Docking

The native and mutant FLT3 protein structures were first prepared by removing water molecules. Subsequently, the structures were converted to the Pdbqt format using AutoDock in preparation for docking. Docking analysis was executed using AutoDock Version 4.2 [29] in conjunction with ADT Tools 1.5.6. Intermediate steps, including energy minimization for protein and ligand structures in the Pdbqt format and grid box generation, were handled using the graphical user interface of AutoDock Tools. AutoDock added polar hydrogens, Kollman atomic charges, solvation parameters, and fragmental volumes to the protein. The prepared structures were saved in Pdbqt format. For grid map file generation, AutoGrid was employed, utilizing a grid box with dimensions set to 60 × 60 × 60 points in x, y, and z, and a grid spacing of 0.375 Å. The grid box center was adjusted based on the position of the co-crystal ligand. AutoDock’s iterative local search global optimizer was used to generate protein–ligand poses. Complexes with the lowest binding free energy (greater negative ΔG values) were selected as the starting structures for molecular dynamics (MD) simulations. In total, nine complexes, namely native-Quizartinib, Y842C-Quizartinib, Y842F-Quizartinib, native-Sorafenib, Y842C-Sorafenib, Y842F-Midostaurin, native-Midostaurin, Y842C-Midostaurin, and Y842F-Midostaurin, were chosen as initial structures for MD simulations.

### 2.3. MD Simulations

The topologies for both ligand and protein structures were generated using the PRODRG server [30] and the editconf script from the GROMACS software (version 4.6.7), respectively. The protein topologies were derived using the GROMOS96 43a1 force-field [31]. Subsequently, ligand topologies were combined with protein topologies to create a protein–ligand complex. This complex was situated inside a cubic box populated with the simple point charge (SPC) water model. To neutralize the system, counter ions (Na^+^ and Cl^−^) were introduced. The neutralized system then underwent 50,000 steps of energy minimization using the steepest descent algorithm. Position restraints for the ligand and temperature coupling groups were established at this juncture. The energy-minimized systems proceeded to a two-phase equilibration, each spanning 1000 ps. The initial phase operated within an isothermal–isochoric ensemble, ensuring a constant number of particles, volume, and temperature. This step aimed to stabilize the system’s temperature. In the subsequent phase, the system’s pressure and density were equalized under the isothermal–isobaric ensemble, maintaining a constant number of particles, pressure, and temperature. The temperature and pressure during these ensembles were regulated by the velocity rescaling thermostat [32] and the Parrinello–Rahman barostat [33], respectively. Following equilibration, all position restraints were released, and the systems were subjected to 1000 ns MD simulations. These MD trajectories facilitated the calculation of thermodynamic binding free energies through the Molecular Mechanics-Poisson Boltzmann Surface Area (MM-PBSA) method.

### 2.4. MM-PBSA Calculations

We selected the last 50 ns of the most stable trajectories from MD simulations to compute the binding free energies of protein–ligand systems using the g_mmpbsa tool [34]. This tool synergizes binding energy calculations with high-throughput MD simulations, accounting for conformational changes that occur during protein–ligand binding. While the method does not compute the entropic terms, it is ideal for comparing the relative binding energies of molecules that interact within the same binding pocket.

The binding free energy for protein–ligand, protein–protein, protein–DNA complexes, or any biomolecular assemblage can be theoretically expressed by the equation:

ΔG_binding_ = G_complex_ − (G_protein_ + G_ligand_) (1)

Each component in Equation (1) can further be defined by:

G_x_ = (E_MM_) − TS + (G_solvation_) (2)

In this equation, ‘x’ can represent G_complex_, G_protein_, or G_ligand_. EMM stands for the average molecular mechanics potential energy in a vacuum. The term TS symbolizes the entropic contribution to free energy in a vacuum, with ‘T’ and ‘S’ denoting temperature and entropy, respectively. Lastly, Gsolvation refers to the free energy of solvation.

### 2.5. Drug Sensitivity Assays

The Ba/F3 cell line was procured from Deutsche Sammlung von Mikroorganismen und Zellkulturen (DSMZ, Braunschweig, Germany). The cells were cultured in RPMI 1640 medium supplemented with 10% heat-inactivated fetal bovine serum (FBS) (Thermo Fisher Scientific, Waltham, MA, USA), 100 U/mL penicillin, 100 µg/mL streptomycin (Corning, Corning, NY, USA), and 10 ng/mL murine IL3 (Thermo Fisher). All inhibitors were sourced from MedChemExpress, Monmouth Junction, NJ, USA. Ba/F3 cells, after being stably transfected with FLT3-ITD and activation loop tyrosine mutants, were maintained in the same medium as the parental Ba/F3 cells. For the drug sensitivity assays, 10,000 cells were seeded in IL3-free medium and exposed to ten distinct drug concentrations, ranging from 5 picomolar to 10 micromolar, for 72 h. Cell viability was then assessed post the 72 h period using CellTiter-Glo (Promega, Madison, WI, USA).

### 2.6. Apoptosis Assay

Ba/F3 cells stably expressing FLT3-ITD, or activation loop mutants were treated with various drug concentrations for 48 h. Following treatment, the cells were processed, and apoptotic cells were quantified using the FITC-Annexin-V/7-AAD kit (BD Biosciences, Franklin Lakes, NJ, USA) as per the manufacturer’s instructions.

### 2.7. ConPLex Analysis

The kinase domain of FLT3 was identified using the NCBI’s Conserved Domains Search. For our analysis, we retrieved the Simplified Molecular Input Line Entry System (SMILES) notations of selected small molecules from the ChEMBL Database. To simulate mutations, the Y842 residue in FLT3 was replaced with both Cysteine (C) and Phenylalanine (F). Using a custom Python script, these modified FLT3 sequences were combined with the small molecules’ SMILES notations, resulting in more than 5.7 million protein–small molecule pairs. These pairs were then evaluated using the pre-trained ConPLex (conplex-dti 0.1.10) model [24] to predict interaction scores, providing insight into potential binding affinities between the FLT3 variants and the small molecules.

### 2.8. Xepto50

Xepto50 (version 0.0.2) is designed to handle data ranging from a single experiment to multiple experiments, encompassing various cell lines and drugs, all within a single Excel file. The software intelligently detects the number of response columns. When there are two or more response columns, Xepto50 calculates using the mean response for subsequent analyses. If there are three or more response columns, the software not only plots the standard error of the mean (SEM) but also provides functionality to compute and remove outliers. Xepto50 is versatile in its data input capabilities; it can accept response data in the form of viability or inhibition, whether presented as a ratio or a percentage. However, for consistency and ease of analysis, it internally converts all input responses to a format that represents inhibition in percentage terms. For curve fitting and analysis, Xepto50 applies a four-parameter logistic regression function.

$$Response={response}_{min}+\frac{{response}_{max}-{response}_{min}}{1+10^{hillslope({log}_{10}\left({IC}_{50}M\right)-{log}_{10}\left(drug\;concentration\;(M)\right))}}$$

Xepto50 offers an integrated solution for analyzing drug response experiments. Initially, the tool employs the curve_fit function from scipy.optimize to fit the data. To further refine this fit, the lmfit model is subsequently utilized. In terms of metrics, Xepto50 is equipped to calculate traditional IC_50_, interpolated IC_50_, and area under the curve (AUC). Additionally, it determines drug sensitivity scores, DSS1, DSS2, and DSS3. Of note is the unique “Xepto50 score” introduced by the software. This score is derived by determining the AUC between the interpolated IC_50_ and the sum of the interpolated IC_50_ and a constant value. The baseline response value used for this calculation is 50. The result is then normalized by dividing it by the total area spanning between the IC_50_ and the aforementioned sum of the interpolated IC_50_ and the constant value.

Ensuring data quality and reliability is of utmost importance. To that end, Xepto50 offers a comprehensive suite of quality scores, including R^2^ Score, Adjusted R^2^ Score, standard error of the estimate (Sy.x), root mean squared error (RMSE), Shapiro–Wilk normality test *p*-value, explained variance score, maximum residual error, root mean absolute error (RMAE), and mean absolute percentage error (MAPE), among others. For user accessibility, Xepto50 features a user-friendly Graphical User Interface (GUI). This ensures a seamless experience even for individuals who may not be versed in programming. The tool is also designed for easy setup within a conda environment. Installation is straightforward: pip install xepto50. Once installed, users can initiate the software by simply entering the command xepto50.

## 3. Results

### 3.1. Identification of FLT3 Interacting Small Molecules Using Molecular Contrastive Learning and Protein Language

**Figure 1:**
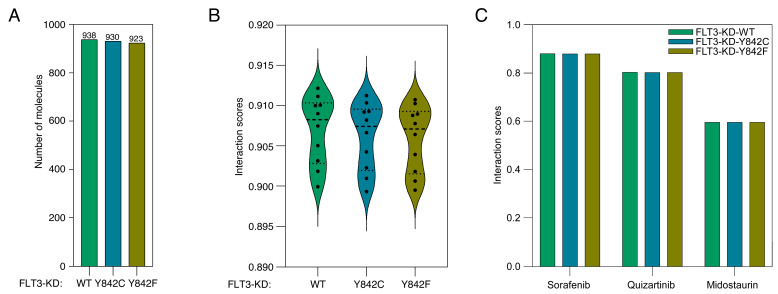
Interaction dynamics of FLT3 kinase domain and its mutants with small molecules. (**A**) Distribution of interaction scores for over 5.7 million unique FLT3-KD-small molecule pairs sourced from the ChEMBL database. ConPLex platform with a threshold score of >0.8 was used to identify interactions between small molecules and FLT3 kinase domains. (**B**) Trend analysis of interaction scores, revealing a descending order from FLT3-KD > FLT3-KD-Y842C > FLT3-KD-Y842F. (**C**) Specific interaction scores for FLT3 inhibitors.

Molecular contrastive learning is an emerging technique that has garnered significant attention due to its ability to leverage vast datasets of small molecules for probing molecular interactions. A novel integration of this methodology with protein language modeling was observed in a recent publication [24]. For our analysis, we harnessed an expansive dataset of more than 1.9 million small molecules sourced from the ChEMBL database. These were juxtaposed with the FLT3 kinase domain (FLT3-KD) and its mutants, Y842C and Y842F. Consequently, the analysis involved over 5.7 million unique FLT3-KD-small molecule pairs. Employing a pretrained model within the ConPLex platform, we discerned that 938 small molecules manifested interactions with the FLT3-KD, contingent on a ConPLex interaction score threshold of >0.8. Adopting an identical score threshold, we identified interactions of 930 small molecules with FLT3-KD-Y842C and 923 molecules with FLT3-KD-Y842F (Table S1 and Figure 1A). The vast majority of the 930 small molecules from the ChEMBL database are experimental compounds and do not have assigned common names. Only 18 of these molecules are currently recognized by a specific name. Interestingly, while the interaction scores exhibited no significant statistical divergence between the wild-type FLT3-KD and its mutants, an observable trend emerged. The interaction scores consistently descended in the order of FLT3-KD > FLT3-KD-Y842C > FLT3-KD-Y842F (Figure 1B). This trend insinuates that mutations within the activation loop could potentially modulate the interaction dynamics between inhibitors and the FLT3 kinase domain. Furthermore, the specific characteristics of these mutations may influence the nature of these interactions in distinct ways. Notably, established FLT3 inhibitors like quizartinib, ponatinib, and Sorafenib all had interaction scores surpassing 0.8. Recently, Quizartinib received FDA approval for use in newly diagnosed AML when administered alongside chemotherapy [9]. Although Sorafenib is currently approved for solid tumors, it has demonstrated significant promise in AML treatment and may be considered for clinical approval in this context [35]. Midostaurin, which was the first kinase inhibitor to obtain FDA approval for AML [36], displayed a score below 0.6 (Figure 1C). Despite this, due to its clinical significance, we included Quizartinib, Sorafenib, and Midostaurin in our further analyses. Gilteritinib, another kinase inhibitor approved by the FDA for AML [37], had a score below 0.2; hence, it was excluded from subsequent examination.

### 3.2. Binding Free Energy Analysis of Native and Mutant FLT3 Structures with Drug Molecules Using the MM-PBSA Approach

As we observed a trend in ConPLex interaction scores where mutants displayed slightly compromised interactions, we wanted to use structure-based approaches to measure the effect of point mutations. We have selected three inhibitors: Quizartinib, Sorafenib, and Midostaurin, due to their wide use in FLT3 research. We utilized the MM-PBSA method to compute thermodynamic binding free energies for both native and mutant FLT3 structures interacting with various drug molecules. The native FLT3 protein structure was sourced from the PDB database [26]. We introduced point mutations at position Y842 to create models of the Y842C and Y842F mutant structures. The kinase domain of the native experimental structure, in complex with the inhibitor quizartinib, was chosen as the binding site for our free energy calculations. We docked the molecules Quizartinib, Sorafenib, and Midostaurin onto the specified binding pocket of the native and mutant FLT3 structures. The docked complexes exhibiting the most stable conformations underwent MD simulations, followed by thermodynamic binding free energy calculations (Table 1). The root mean square deviation (RMSD) and radius of gyration (Rg) for native FLT3 and mutated protein structure complexes with Quizartinib, Sorafenib, and Midostaurin had shown stable trajectories throughout the simulation period (Figures S2 and S3). A significant amount of hydrogen bonding was also observed for native FLT3 and mutated protein structures with the respective drug molecules (Figure S4). Over time, the MM-PBSA method has gained traction and is now a recognized approach for predicting and comparing the binding free energies of various biomolecular structures [38,39,40,41]. Binding free energy inversely relates to the affinity between proteins and ligands. Our analyses revealed that mutations in FLT3 structures influenced the binding free energies. Specifically, the binding free energy dropped for both mutant FLT3 proteins when interacting with Sorafenib, compared to the native FLT3–Sorafenib complex. It is also evident from the h-bond analysis that in case of mutant–Sorafenib complexes very consistent pattern of hydrogen bonding was observed (Figure S4) In contrast, with Midostaurin, the binding free energy for mutant structures was higher than for the native protein complex. Intriguingly, Quizartinib presented intermediate binding energy levels in both native and mutant structures. The van der Waals energy was the most significant contributor to overall binding free energy. However, with midostaurin, electrostatic energy had a more favorable contribution in both mutant structures compared to the van der Waals energy. The polar solvation energy component contributions were generally unfavorable for the total binding free energy across all protein–ligand complexes.

### 3.3. Differential Apoptotic Responses in FLT3-ITD Expressing Ba/F3 Cells Harboring Y842 Mutations

**Figure 2:**
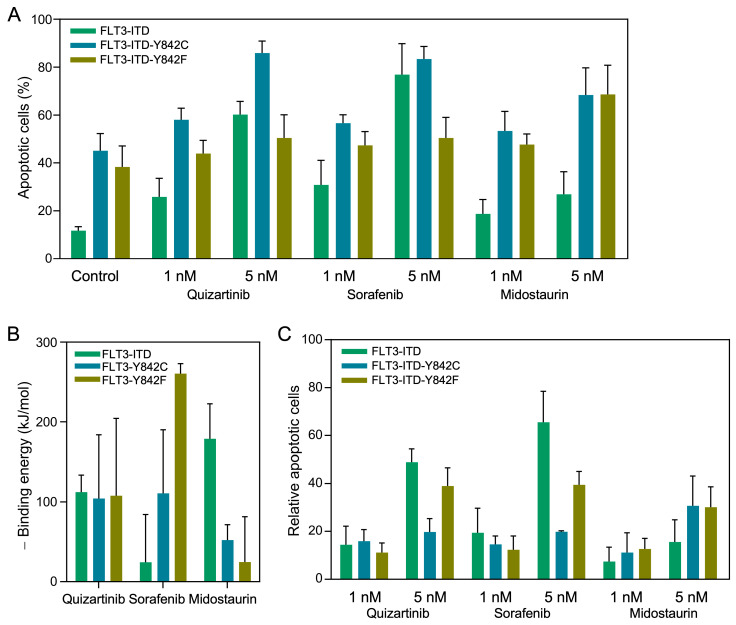
Differential apoptotic responses and binding energy analyses of FLT3-ITD Y842 mutants under drug treatments. (**A**) Measurement of apoptotic cells using the annexin V-7-AAD kit after treating cells with specific inhibitors for 48 h prior to processing and analysis (*n* = 5). (**B**) Binding energy, represented as negative values, is plotted against various drug–mutant pairs. (**C**) Calculation of relative apoptotic cells by subtracting the number of apoptotic cells observed in DMSO-treated controls from those treated with specific inhibitors.

We next aimed to compare the apoptosis responses among different Y842 mutants. We have previously demonstrated that the murine Interleukin 3 (IL3)-dependent 32D cell line harboring the FLT3-ITD-Y842F mutation exhibits comparatively decreased viability and increased apoptosis when compared to cells containing the FLT3-ITD mutation upon withdrawal of IL3 [11]. In this study, we generated Ba/F3 cells that stably express FLT3-ITD, FLT3-ITD-Y842F, and FLT3-ITD-Y842C. These cells were cultured in the presence of murine IL3, but we removed IL3 prior to adding drugs for the apoptosis assays. Cells were treated with either 1 nM or 5 nM of Quizartinib, Sorafenib, Midostaurin, or the equivalent volume of DMSO, which was used to prepare the drug solutions. Our observations revealed that while the expression of FLT3-ITD alone was sufficient to support the survival of Ba/F3 cells in the absence of IL3, cells expressing FLT3-ITD alongside Y842F or Y842C mutations had approximately four times more apoptotic cells (as shown in Figure 2A). Regardless of the Y842 mutations, the treatment with inhibitors enhanced the apoptosis response. Given that different drug–mutant combinations showed varied binding energies (Figure 2B), we calculated the relative apoptosis by subtracting the number of apoptotic cells in the DMSO-treated samples from the total (Figure 2C). A similar trend was observed in the samples treated with Quizartinib and Sorafenib, whereas an opposite trend was evident in the Midostaurin-treated samples (Figure 2C). This finding underscore the role of the Y842 mutations in modulating apoptotic responses in Ba/F3 cells expressing FLT3-ITD. Specifically, cells harboring FLT3-ITD alongside Y842F or Y842C mutations demonstrated a heightened apoptotic response, approximately four-fold greater, in comparison to cells expressing only FLT3-ITD. This suggests that the presence of these mutations may render cells more susceptible to apoptosis in the absence of IL3. Interestingly, while drug treatment amplified apoptosis across the board, different drug–mutant combinations exhibited varied responses. The differential binding energies observed for each drug–mutant pair may offer insights into the mechanistic differences in drug efficacy and specificity. Importantly, while Midostaurin followed the general trend, Sorafenib behaved oppositely. This highlights the nuanced interplay between specific mutations and drug treatments, emphasizing the need for personalized therapeutic strategies in targeting FLT3-ITD associated malignancies.

### 3.4. Evaluation of Drug Sensitivity Metrics and the Introduction of the Xepto50 Scoring System for Enhanced Drug Efficacy Analysis

**Figure 3:**
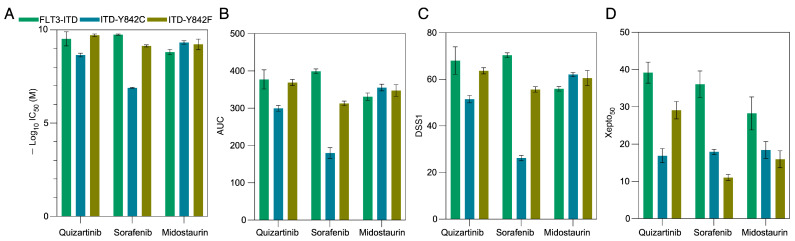
Assessment of various drug sensitivity metrics. (**A**) IC_50_ values were determined using GraphPad Prism 9 (*n* = 5), derived from interpolated values at 50 and subsequently transformed to a negative log_10_ scale. (**B**) The area under the curve (AUC) was computed from the same dataset, with a baseline response set at 10. (**C**) Drug Sensitivity Score 1 (DSS1) was determined using the Xepto50 software (version 0.0.2). (**D**) The Xepto50 score was derived from the normalized AUC at a specific interval on a logarithmic concentration axis.

As apoptotic responses demonstrated a partial correlation with in silico data, our subsequent objective was to assess cell viability to determine drug sensitivity indices. Initially, we quantified the interpolated IC_50_ by employing a four-parameter logistic curve-fit model (Figure S5A) and area under the curve (AUC) employing GraphPad Prism 9. Notably, there were no significant disparities in terms of IC_50_ (represented as −log_10_IC_50_, Figure 3A) or AUC (Figure 3B), with the exception that the Y842C mutant exhibited reduced responsiveness to Sorafenib. To further assess various metrics, we introduced Xepto50, a robust tool capable of determining IC_50_, interpolated IC_50_, AUC, and drug sensitivity scores (DSS1, DSS2, and DSS3) in batch mode from an Excel file input. Xepto50, a Python-based application with a graphical user interface (GUI), exhibited interpolated IC_50_ and AUC values consistent with those of GraphPad Prism 9 (Figure S5B,C). Additionally, the trends observed in DSS1 (Figure 3C), DSS2 (Figure S5D), and DSS3 (Figure S5E) paralleled those of IC_50_ and AUC metrics, implying that these drug sensitivity metrics might not fully encapsulate theoretical observations. The four-parameter logistic regression curve remains a prevalent model for gauging drug sensitivity. A lateral shift in this curve denotes reduced potency (Figure S5E), whereas a diminished slope indicates compromised cooperativity (Figure S5F). Conversely, a vertical shift of the maximum value alludes to heightened efficacy (Figure S5G). Beyond these, multiple other curve manifestations can be discerned (Figure S5H–J). Given that a drug’s impact is an amalgamation of these factors, deriving conclusions from a singular parameter could obscure true drug efficacy. For instance, drugs with identical IC_50_ values might display stark differences in cooperativity and efficacy (Figure S5G,H). However, a perusal of the logistic regression curve could elucidate these nuances. It is crucial to underline that a drug exhibiting low potency might be highly efficacious at elevated concentrations, a nuance potentially overlooked by prevailing scoring techniques. Thus, we advocate for an alternative metric—the Xepto50 score—that gauges the normalized area under the curve at the 50% interpolated value within a specified range. Distinctly, the Xepto50 score remains unaffected by the logistic regression curve’s position but is acutely responsive to its shape, rendering it ideal for discerning drug efficacy. Importantly, our findings revealed that the Xepto50 score better mirrors apoptosis response and theoretical values (Figure 3D).

## 4. Discussion

The advancements in molecular modeling, combined with the rise of machine learning in drug discovery, are poised to bring transformative changes to pharmacology. Among these innovations, molecular contrastive learning stands out as a burgeoning technique, demonstrating its aptitude in deciphering vast molecular interactions with remarkable accuracy. In line with findings from prior studies [24], our research capitalizes on the extensive dataset of small molecules sourced from ChEMBL, shedding light on interactions within the FLT3 kinase domain. We observed a distinct trend in interaction scores, descending in the sequence of FLT3-KD > FLT3-KD-Y842C > FLT3-KD-Y842F. This pattern indicates that mutations within the activation loop might be instrumental in altering inhibitor interactions with the FLT3 kinase domain. Additionally, the protein language model discerned variations resulting from amino acid alterations in the protein sequences. Given the established knowledge that protein mutations can profoundly impact therapeutic outcomes [1,11,16], it is crucial to recognize and comprehend these nuanced genetic shifts when considering therapeutic strategies.

The RMSD trajectories from MD simulations for native and mutant protein complexes with three drug molecules remain converged throughout the simulations and suggested the stability of the complexes. Thereafter, all the complexes were subjected to MM-PBSA analysis. Moreover, the application of the MM-PBSA method, a widely acknowledged technique, reaffirmed the impact of point mutations on binding free energies [42,43]. The variable free energy readings between native and mutant structures, in the presence of different inhibitors, might elucidate some mechanistic underpinnings of the observed efficacy differences. This could help inform inhibitor selections based on specific mutation profiles. Furthermore, our empirical findings in Ba/F3 cells highlighted the functional implications of the Y842 mutations. Their increased apoptotic responses, especially in the absence of IL3, suggest that these mutations might render the cells more vulnerable to therapeutic interventions. These data further advocate for the development of personalized therapeutic regimes. Drug-specific responses, especially the contrasting behavior of Midostaurin and Sorafenib, serve as an important reminder of the intricate and multifaceted interactions between drugs and their molecular targets.

Apart from the established theoretical values, our exploration into comparing drug sensitivity both at apoptosis and viability levels unveiled some inconsistencies with theoretical predictions. Specifically, while Quizartinib and Midostaurin exhibited higher congruence with theoretical values, the cellular response to Sorafenib did not align with its predicted theoretical binding energy. This disparity may either highlight the limitations of our theoretical models or suggest that Sorafenib interacts at different sites within the kinase domain, especially given that we utilized the Quizartinib association site for docking.

Moreover, our findings indicate that traditional drug sensitivity metrics might not consistently represent real-world outcomes. The assessment of drug sensitivity metrics, punctuated by the introduction of the Xepto50 scoring system, has addressed a longstanding challenge in drug discovery. Although widely used metrics like IC_50_ provide invaluable perspectives, they occasionally miss capturing the entire spectrum of drug efficacy. This gap becomes pronounced in situations where drugs have similar IC_50_ values but divergent mechanisms of action. Given the Xepto50 score’s emphasis on curve shapes rather than mere positions, it promises a more comprehensive insight into drug mechanisms. By leveraging such advanced metrics, the drug development process could be refined, paving the way for therapies that are both potent and adaptive to diverse mechanisms of action.

### 4.1. Limitations

The study encountered several limitations, starting with the ConPLex platform’s inefficiency in accurately scoring certain clinically relevant FLT3 inhibitors like Gilteritinib and Midostaurin, indicating the need for improvements in the pretrained model. Discrepancies between theoretical predictions and actual cellular responses to drugs also pointed to potential shortcomings of the computational approaches adopted. One contributing factor could be the uniform docking strategy employed for all inhibitors, where a flexible approach might yield more accurate results. Additionally, the complex biological interplay observed in wet lab experiments, including off-target activities of kinase inhibitors, could further explain the variance from theoretical expectations. The study’s reliance on traditional drug sensitivity metrics such as IC_50_, while standard, may not adequately reflect the intricacies of drug efficacy. This is especially evident in scenarios where drugs with similar IC_50_ values have diverse mechanisms of action, underscoring the limitations of using IC_50_ values as the sole measure of drug effectiveness. Although the Xepto50 scoring system represents progress in addressing these issues, it is not without its potential shortcomings, which require more extensive investigation. The Xepto50 metric, being relatively new, has aspects of drug response it might not cover, and its comparative effectiveness remains to be fully assessed. Moreover, extrapolating the empirical findings from Ba/F3 cells, particularly regarding Y842 mutations, to other cellular contexts or in vivo conditions, is not straightforward, highlighting the divide between laboratory and clinical settings. These limitations underline the importance of future research directed at refining computational models, developing alternative drug sensitivity metrics, expanding the range of genetic mutations studied, and bridging the gap between in vitro results and clinical applications.

### 4.2. Advantages and Disadvantages

The study presents a comprehensive approach to drug discovery in the context of AML, specifically targeting FLT3 activation loop mutants, utilizing the ConPLex platform for a large-scale analysis of millions of inhibitor combinations. The application of advanced computational tools, such as molecular contrastive learning and protein language modeling, provided a deep dive into the interaction dynamics between inhibitors and the FLT3 structure. A significant advantage of this research is the introduction of the Xepto50 scoring system, which offers a refined metric for assessing drug efficacy. This is particularly beneficial when traditional metrics like IC_50_ fall short of capturing the intricate effects of drug interactions. Furthermore, the study extends its relevance through empirical assessments, linking structural insights to cellular responses under drug treatments. However, the research also acknowledges its limitations, such as the less-than-optimal scoring of certain clinically relevant FLT3 inhibitors by the pretrained ConPLex model. The discrepancy observed between theoretical predictions and cellular responses signals a potential gap in the models used, suggesting a need for more adaptable docking strategies and consideration of the complex nature of kinase inhibitors’ off-target activities. The focus on specific mutations, primarily the Y842 mutations, may not encompass the full spectrum of genetic variations pertinent to FLT3-ITD malignancies. Moreover, the promising new Xepto50 scoring system still requires further validation, and there is a recognized challenge in extrapolating in vitro findings to in vivo conditions and clinical effectiveness. Thus, while the study may contribute to the areas of FLT3 research, it also paves the way for future work to refine computational models, broaden the scope of genetic mutations studied, and better translate laboratory findings into clinical therapies.

## 5. Conclusions

In conclusion, our findings underscore the potential of leveraging advanced molecular modeling techniques, reinforced with empirical validations, to enhance our understanding of drug–target interactions. The discerning insights obtained from such analyses, when combined with innovative metrics like Xepto50, can pave the way for more informed and effective therapeutic strategies. Future studies could further delve into the mechanistic intricacies of these interactions, potentially revealing novel therapeutic targets or strategies to combat FLT3-ITD-associated malignancies.

## Figures and Tables

**Table 1:** The thermodynamic binding free energy and its constituents calculated by the MM-PBSA approach.

FLT3 Protein	FLT3 Inhibitors	van der Waals Force (kJ/mol)	Electrostatic Energy (kJ/mol)	Polar Solvation Energy (kJ/mol)	SASA Energy (kJ/mol)	Binding Energy (kJ/mol)
Native	Quizartinib	−190.70 ± 15.48	−110.75 ± 24.56	206.74 ± 34.49	−17.72 ± 1.28	−112.45 ± 21.08
	Sorafenib	−42.12 ± 78.17	−7.15 ± 14.10	28.66 ± 48.37	−3.65 ± 7.00	−24.27 ± 60.15
	Midostaurin	−326.70 ± 49.07	−199.75 ± 40.48	372.96 ± 58.41	−25.37 ± 3.19	−178.85 ± 43.74
Mutant I (Y842C)	Quizartinib	−258.69 ± 74.61	−12.69 ± 29.22	186.68 ± 26.86	−19.79 ± 3.54	−104.49 ± 79.30
	Sorafenib	−140.86 ± 107.91	−13.04 ± 16.37	54.82 ± 53.67	−11.69 ± 6.83	−110.76 ± 77.65
	Midostaurin	−116.65 ± 66.32	−132.48 ± 106.74	209.11 ± 177.76	−12.02 ± 6.89	−52.04 ± 19.61
Mutant II (Y842F)	Quizartinib	−179.77 ± 134.54	−53.30 ± 43.73	139.41 ± 109.23	−14.19 ± 9.53	−107.85 ± 96.60
	Sorafenib	−329.85 ± 12.03	−70.41 ± 9.66	162.84 ± 11.38	−23.24 ± 0.87	−260.67 ± 12.46
	Midostaurin	−120.79 ± 113.89	−121.86 ± 115.33	228.13 ± 196.64	−10.12 ± 9.78	−24.643 ± 57.03

## Data Availability

The Python library, Xepto50, can be obtained from the Python Package Index (PyPI) at www.pypi.org/project/xepto50/ (accessed on 5 November 2023), or from the GitHub repository at www.github.com/kazilab/xepto50 (accessed on 5 November 2023). For raw data, please send a request to J.U.K.

## Supplementary Materials

The following supporting information can be downloaded at: https://www.mdpi.com/article/10.3390/cancers15225426/s1, Figure S1: Methodology flowchart; Figure S2: Time evolution of backbone RMSDs; Figure S3: The radius of gyration; Figure S4: Hydrogen bonding profile; Figure S5: Drug sensitivity scores; Table S1: ConPLex scores with a cut-off of 0.8.
