# Supplementary material for: Understanding the Role of Activation Loop Mutants in Drug Efficacy for FLT3-ITD
Source: Cancers (Basel). 2023 Nov 15;15(22):5426. doi: 10.3390/cancers15225426 (PMC10670458; doi:10.3390/cancers15225426)
Supplement: Supplementary file 1 [file cancers-15-05426-s001.zip › cancers-2686709-supplementary.pdf]

Figure S1. Methodology flowchart. This flowchart provides a concise overview of the sequential methodological steps used to investigate the interactions between FLT3-Y842 mutants and various inhibitors. It outlines the progression from ConPLex analysis through molecular dynamics (MD) simulation and docking, followed by apoptosis assays and cell proliferation tests, concluding with the development of the Xepto50 application to address the limitations of IC50 for drug efficacy evaluation.

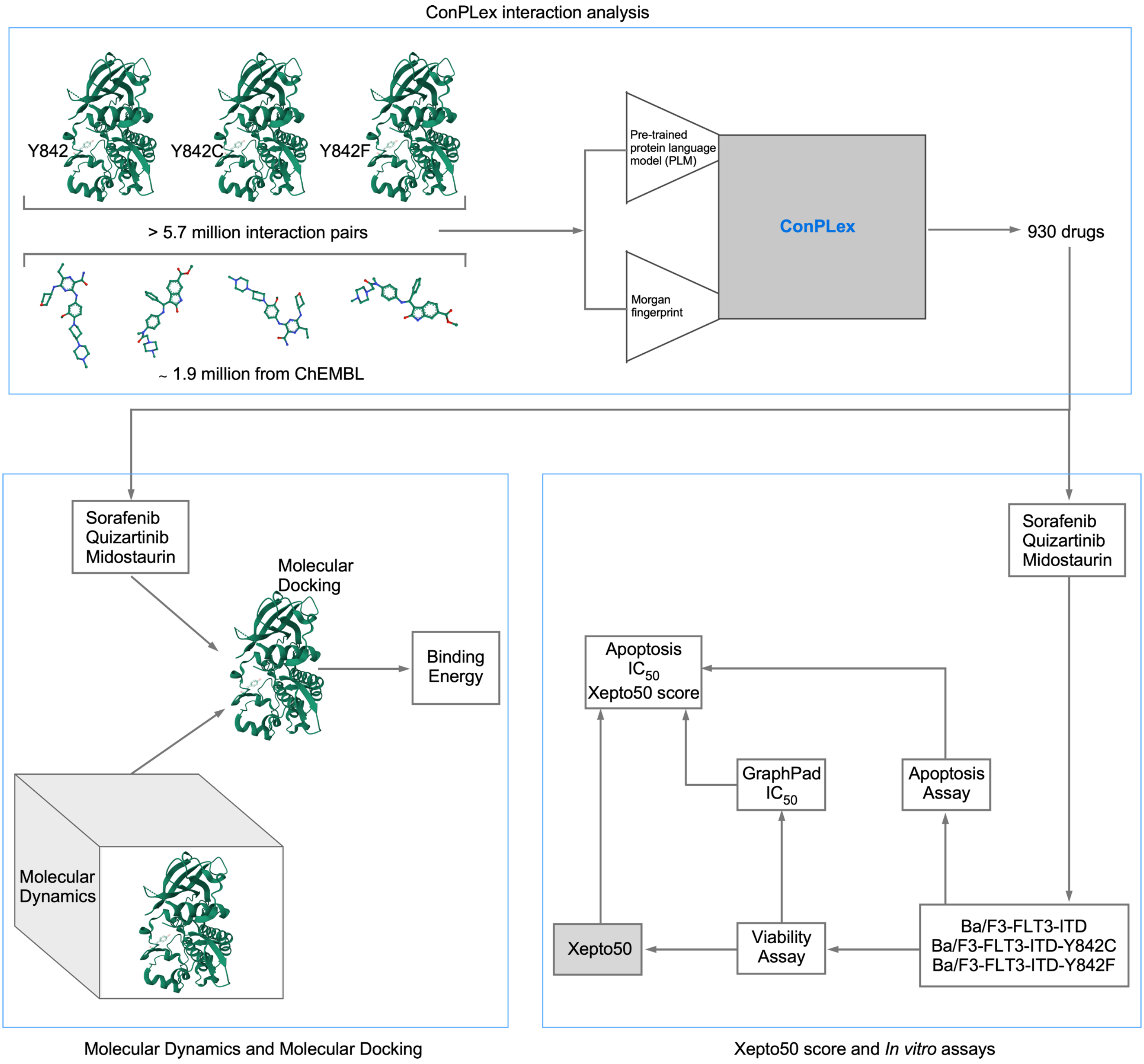

Figure S2. Time evolution of backbone RMSDs is shown as a function of time for native FLT3 and mutant (Y842C and Y842F) protein complexes with midostaurin, sorafenib, and quizartinib.

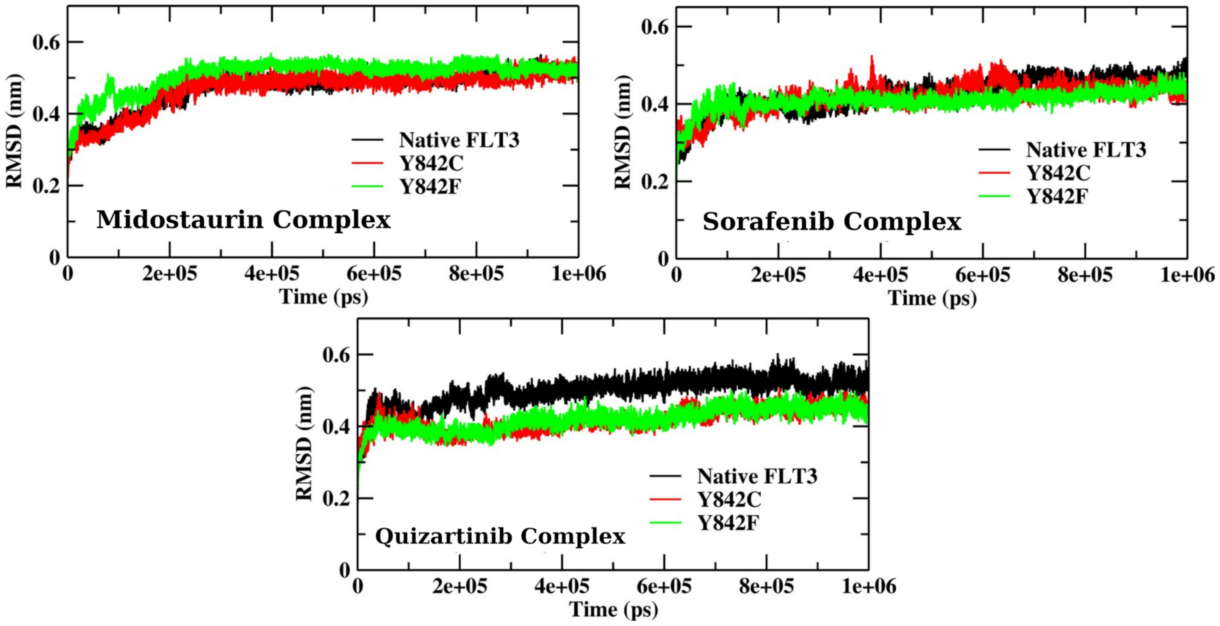

Figure S3. The radius of gyration (Rg) for native FLT3 and mutant (Y842C and Y842F) protein complexes with midostaurin, sorafenib, and quizartinib.

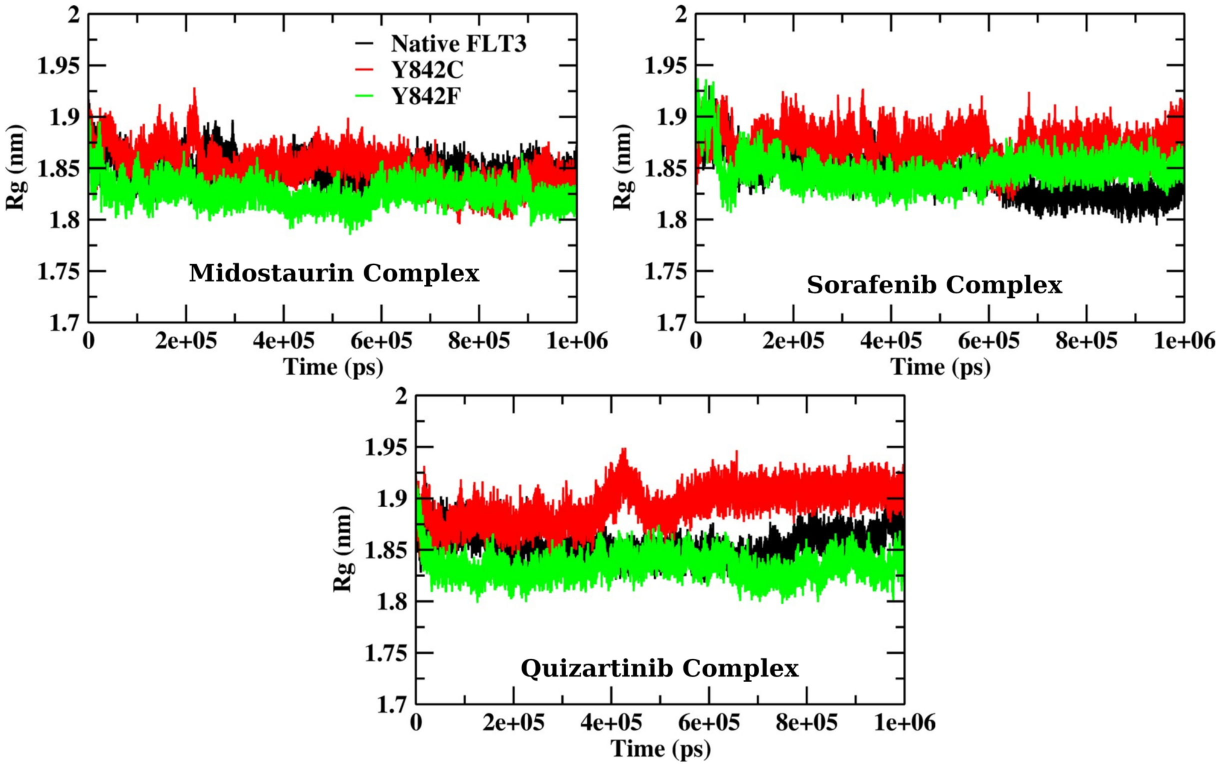

Figure S4. Hydrogen bonding profile for native FLT3 and mutant (Y842C and Y842F) protein complexes with midostaurin, sorafenib and quizartinib.

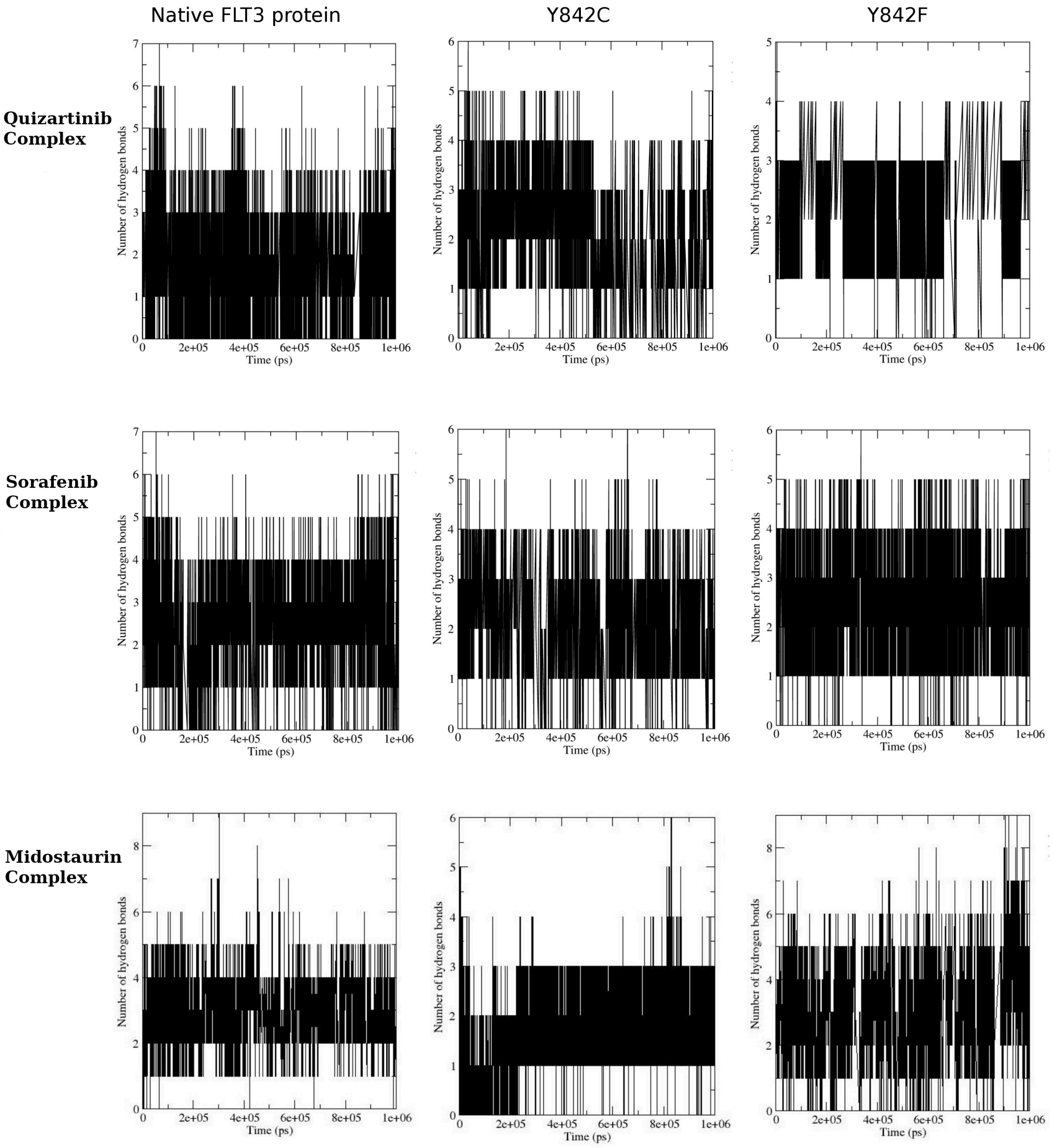

Figure S5. Drug sensitivity scores. (A) Four-parameter logistic regression curves plotted using GraphPad Prism 9. (B-E) Drug sensitivity metrics including interpolated IC50 (B), area under the curve (C), as well as drug sensitivity scores DSS2 (D) and DSS3 (E), were determined using the Xepto50 application. (F-K) Sample plots were produced using Python scripts.

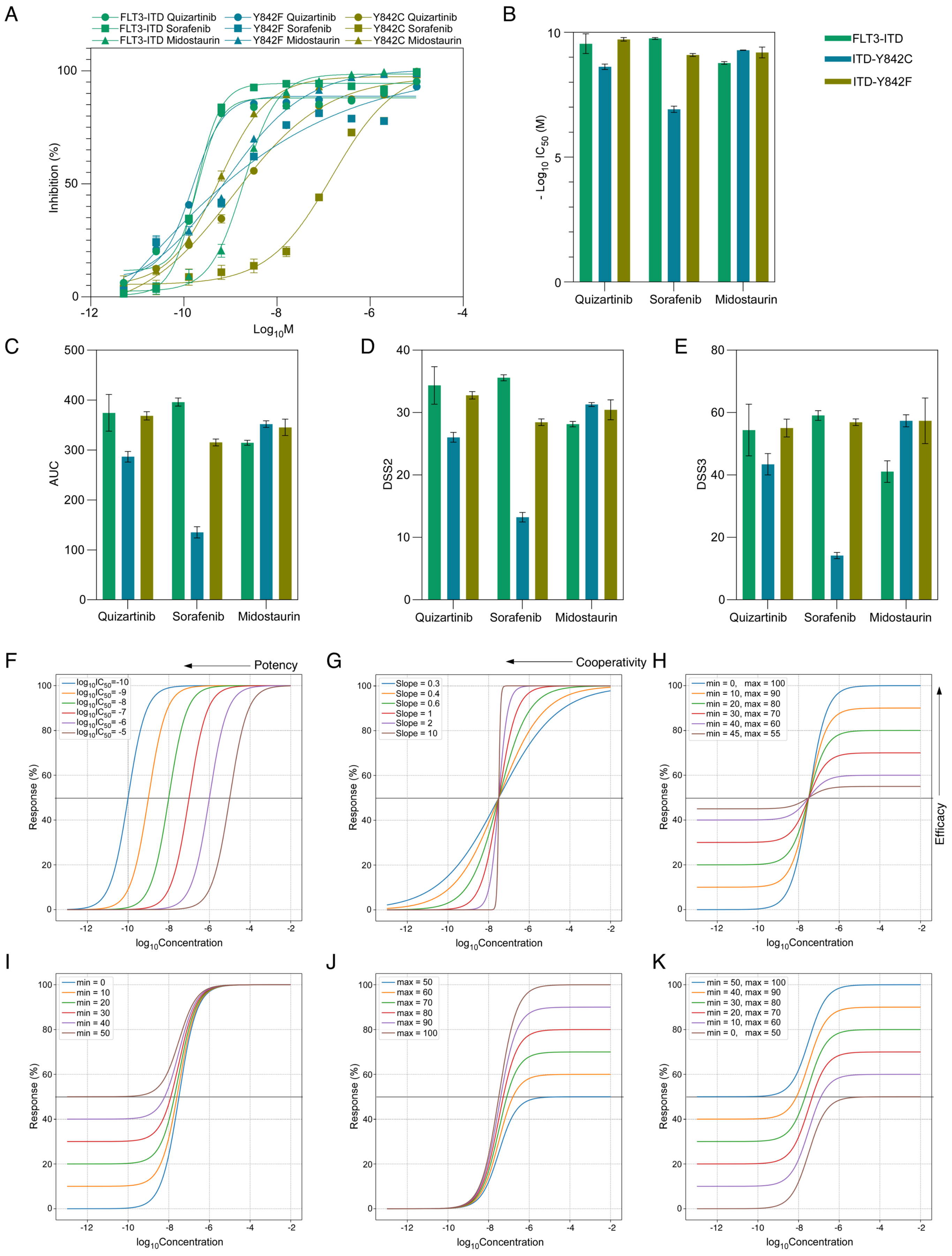

Table S1. ConPLex scores with a cut-off of 0.8.

ChEMBL ID	FLT3-KD	FLT3-KD-Y842C	FLT3-KD-Y842F	Known Name
CHEMBL1336	0.88009953	0.879559	0.8794492	SORAFENIB
CHEMBL1760433	0.88009953	0.879559	0.8794492	[11C]-SORAFENIB
CHEMBL522892	0.86647534	0.8659795	0.86576295	DOVITINIB
CHEMBL2029988	0.85907567	0.85821617	0.85826945	CEP-32496
CHEMBL1721885	0.85760915	0.8571367	0.85697377	SU-014813
CHEMBL535	0.83135295	0.8309217	0.83072686	SUNITINIB
CHEMBL382590	0.8186338	0.8182404	0.81800526	ANILINOQUINAZOLINE1
CHEMBL502835	0.81774807	0.8173186	0.8170203	NINTEDANIB
CHEMBL3355009	0.8166779	0.816097	0.8157109	GSK-3179106
CHEMBL587723	0.81602705	0.8156319	0.815433	AEE-788
CHEMBL475251	0.8106434	0.810296	0.8099333	R-406
CHEMBL1171837	0.80931264	0.8084099	0.80808663	PONATINIB
CHEMBL402548	0.8069736	0.80686325	0.80647147	DANUSERTIB
CHEMBL124660	0.80629563	0.8058732	0.8059722	TANDUTINIB
CHEMBL405130	0.8045374	0.80420613	0.8039881	WHI-P131
CHEMBL575448	0.8043259	0.804387	0.80358016	BMS-754807
CHEMBL576982	0.8033969	0.8030422	0.8029254	QUIZARTINIB
CHEMBL3188386	0.8011353	0.8008038	0.80049914	WNT-974
CHEMBL4099649	0.91213614	0.91122913	0.9107297	
CHEMBL1824068	0.911137	0.9103447	0.91026294	
CHEMBL4073468	0.91005933	0.90917754	0.9087718	
CHEMBL4093692	0.9099878	0.9092678	0.9089335	
CHEMBL4083425	0.9089884	0.90818334	0.9077542	
CHEMBL4074024	0.90747494	0.9066489	0.90640724	
CHEMBL4093080	0.9050114	0.90423524	0.903913	
CHEMBL4085324	0.9031414	0.9022602	0.9018226	
CHEMBL4062112	0.90183824	0.900945	0.9006247	
CHEMBL414639	0.8999244	0.8993199	0.8994806	
CHEMBL1824071	0.8997722	0.89911544	0.8990443	
CHEMBL1824298	0.8996656	0.89884377	0.8988637	
CHEMBL4063346	0.8991973	0.89847136	0.89821804	
CHEMBL4083848	0.8985776	0.8977264	0.8973975	
CHEMBL4101411	0.8946892	0.8938756	0.8934852	
CHEMBL4092570	0.89282364	0.891956	0.891501	
CHEMBL3694154	0.89256585	0.8917288	0.8916415	
CHEMBL3694128	0.89222234	0.8916489	0.8918976	
CHEMBL4100308	0.89178735	0.8908453	0.89048016	
CHEMBL168198	0.89102113	0.8905096	0.89049757	
CHEMBL197713	0.8906821	0.8895377	0.8891343	
CHEMBL197715	0.8906821	0.8895377	0.8891343	
CHEMBL593971	0.89053863	0.8899534	0.88947654	
CHEMBL596292	0.89053863	0.8899534	0.88947654	
CHEMBL1821753	0.890119	0.8894323	0.88944614	
CHEMBL4100787	0.8898138	0.8890079	0.8887212	
CHEMBL3651299	0.88862157	0.8877529	0.8870294	
CHEMBL167783	0.8870468	0.8863976	0.88622516	
CHEMBL4072991	0.88703173	0.8865944	0.8864106	

CHEMBL593905	0.8826077	0.88189507	0.88144344
CHEMBL593908	0.8826077	0.88189507	0.88144344
CHEMBL3905242	0.88214403	0.8815531	0.8812837
CHEMBL4080904	0.8812828	0.88060254	0.88026726
CHEMBL3109202	0.87863994	0.8782185	0.8780732
CHEMBL3593316	0.87828445	0.8769963	0.87691456
CHEMBL436362	0.8780625	0.8768972	0.87660456
CHEMBL3109221	0.8777709	0.8771786	0.87713695
CHEMBL3694155	0.8775522	0.8767288	0.87644315
CHEMBL4093872	0.8769698	0.8765542	0.87635064
CHEMBL4070262	0.87691575	0.8762672	0.87591785
CHEMBL4112520	0.8767921	0.87594867	0.8757709
CHEMBL475425	0.87675184	0.8763421	0.87631285
CHEMBL3694129	0.8764372	0.8758733	0.8757378
CHEMBL3923551	0.87599695	0.8755567	0.87532365
CHEMBL3410034	0.8758471	0.8751161	0.8749057
CHEMBL165327	0.8758281	0.8756002	0.87545145
CHEMBL448264	0.87553465	0.87515235	0.8748589
CHEMBL1979690	0.8754481	0.8748603	0.87457025
CHEMBL3979264	0.8753487	0.87477595	0.8746049
CHEMBL3697901	0.8748969	0.87413037	0.8738469
CHEMBL4097446	0.8747295	0.87391865	0.87378794
CHEMBL194352	0.873927	0.8727762	0.8723453
CHEMBL425389	0.873927	0.8727762	0.8723453
CHEMBL4065410	0.8729531	0.872133	0.8716643
CHEMBL3694140	0.8728607	0.8717775	0.8719231
CHEMBL124949	0.87275183	0.87243736	0.8723682
CHEMBL213505	0.8716785	0.8708544	0.8704977
CHEMBL164029	0.87058884	0.8702493	0.8699534
CHEMBL2029992	0.870512	0.8695209	0.8695454
CHEMBL3604921	0.8696372	0.86863625	0.8681123
CHEMBL3780543	0.8693031	0.8692192	0.86891586
CHEMBL1993722	0.8688852	0.86858463	0.8683572
CHEMBL3808854	0.8681216	0.8674141	0.8673032
CHEMBL3896615	0.86811614	0.8675078	0.8670804
CHEMBL3760090	0.8678349	0.867116	0.8668423
CHEMBL45651	0.86743796	0.8669033	0.86626947
CHEMBL95002	0.8672984	0.86633444	0.8659518
CHEMBL1080395	0.8671584	0.8662749	0.86617184
CHEMBL2024463	0.8670567	0.8662722	0.86632586
CHEMBL4286720	0.8666406	0.8663345	0.8661662
CHEMBL1987881	0.86600435	0.86597025	0.86558604
CHEMBL604992	0.8659582	0.86562085	0.86547464
CHEMBL3694141	0.8657173	0.86476374	0.86445516
CHEMBL3099965	0.8654553	0.8652354	0.865039
CHEMBL2029989	0.86517817	0.86438024	0.8644536
CHEMBL3905552	0.8649326	0.8640933	0.86380297
CHEMBL3962233	0.8648173	0.8641703	0.8641095
CHEMBL4176454	0.8644664	0.8641861	0.86406666
CHEMBL13354	0.86429363	0.8638706	0.8635175

CHEMBL159828	0.86403704	0.86361104	0.86322004
CHEMBL196919	0.8631246	0.86330247	0.86308396
CHEMBL2029991	0.8626007	0.8615601	0.8615879
CHEMBL1171086	0.86253166	0.86198753	0.8617922
CHEMBL202370	0.8623625	0.86156493	0.86131513
CHEMBL223583	0.8623167	0.86208963	0.86184907
CHEMBL4171943	0.8623127	0.86189675	0.8616319
CHEMBL3933568	0.86199474	0.8610647	0.860643
CHEMBL520142	0.8619245	0.8620507	0.86181176
CHEMBL1824077	0.861908	0.86147803	0.86138034
CHEMBL179753	0.8616476	0.8607559	0.86058784
CHEMBL3759190	0.861376	0.86073697	0.8603316
CHEMBL3740677	0.86124206	0.86099875	0.86072993
CHEMBL1091191	0.861096	0.86074185	0.86024535
CHEMBL3741983	0.8610452	0.8606995	0.86035204
CHEMBL1081509	0.86085373	0.85997987	0.8597249
CHEMBL223361	0.8608077	0.8604126	0.86002135
CHEMBL102210	0.86067045	0.8600007	0.859855
CHEMBL124089	0.8605256	0.85984194	0.85970604
CHEMBL91167	0.8598268	0.8593034	0.859006
CHEMBL3133751	0.85956556	0.85907096	0.8587807
CHEMBL3604937	0.8595232	0.85889184	0.85872763
CHEMBL31497	0.85937244	0.85872877	0.8584002
CHEMBL214842	0.859287	0.8590081	0.85892123
CHEMBL298679	0.859231	0.85840774	0.8577087
CHEMBL2335881	0.85923	0.85908604	0.8587918
CHEMBL3646724	0.8591881	0.85848033	0.858136
CHEMBL3694157	0.8589844	0.8580996	0.85811406
CHEMBL4103255	0.8585089	0.8579963	0.8574439
CHEMBL522785	0.8580609	0.8580137	0.85761917
CHEMBL1965303	0.85795987	0.85766286	0.857739
CHEMBL4303700	0.85760915	0.8571367	0.85697377
CHEMBL1824293	0.85751605	0.85701096	0.85699284
CHEMBL2335864	0.85745835	0.85726845	0.8569447
CHEMBL381721	0.856797	0.8558206	0.8556872
CHEMBL370655	0.8560351	0.85596395	0.855719
CHEMBL3593283	0.8558384	0.8550383	0.85469663
CHEMBL475424	0.8556198	0.8552033	0.85548687
CHEMBL68401	0.85531473	0.85448843	0.854262
CHEMBL124983	0.8551245	0.85426885	0.8545454
CHEMBL3948354	0.8550103	0.85448456	0.8542873
CHEMBL353138	0.85459477	0.85424083	0.854236
CHEMBL530055	0.8544307	0.8538686	0.85320914
CHEMBL3394472	0.8543712	0.85363317	0.8532463
CHEMBL255819	0.8541709	0.8537041	0.8541029
CHEMBL2029986	0.85407317	0.8534084	0.85342276
CHEMBL3309923	0.8539798	0.8531083	0.8532413
CHEMBL220049	0.8539418	0.85375214	0.85351545
CHEMBL1171045	0.8537357	0.8532312	0.85298234
CHEMBL182515	0.8535418	0.85303336	0.85294354

CHEMBL1824070	0.8533982	0.8524641	0.85262305
CHEMBL3659918	0.8533331	0.8523766	0.8522332
CHEMBL3929290	0.8531787	0.8528007	0.8525908
CHEMBL318804	0.85244566	0.8512039	0.8509942
CHEMBL3758585	0.8523797	0.85167164	0.8514
CHEMBL3410028	0.85233045	0.8517061	0.851298
CHEMBL2029999	0.8521655	0.8518632	0.8517199
CHEMBL3975598	0.8520357	0.851511	0.8511598
CHEMBL3717353	0.8517585	0.8510463	0.85084903
CHEMBL165274	0.8516393	0.85127735	0.8514295
CHEMBL1277253	0.8515424	0.85109806	0.8510293
CHEMBL3742087	0.85146964	0.85067344	0.8501978
CHEMBL593275	0.85112125	0.85069907	0.8503564
CHEMBL3740815	0.8509627	0.8505851	0.850384
CHEMBL414139	0.8503647	0.84940577	0.84898293
CHEMBL206266	0.8503306	0.84979165	0.84956384
CHEMBL2029977	0.8500258	0.84920055	0.84912515
CHEMBL3765767	0.85001314	0.84965956	0.84957826
CHEMBL537328	0.84994704	0.8494994	0.8492348
CHEMBL514806	0.8497515	0.8496845	0.849403
CHEMBL3727725	0.84972274	0.8494243	0.84924436
CHEMBL3732898	0.84969705	0.84911263	0.84858555
CHEMBL3759127	0.8494159	0.8488801	0.8485859
CHEMBL1760034	0.84935474	0.8489707	0.84925306
CHEMBL1081510	0.84933835	0.84878916	0.8487644
CHEMBL340234	0.8491918	0.8487489	0.8488299
CHEMBL1171044	0.84895146	0.8480954	0.84768057
CHEMBL3759830	0.8487274	0.84804946	0.8477669
CHEMBL3781795	0.8486119	0.84880114	0.84839433
CHEMBL374810	0.84851646	0.8481158	0.8478364
CHEMBL4161345	0.848489	0.8482455	0.84816647
CHEMBL1982837	0.8483018	0.84763294	0.84679055
CHEMBL4071974	0.8481417	0.8475443	0.84705645
CHEMBL127771	0.8480828	0.8472739	0.8468962
CHEMBL268731	0.84782517	0.8473944	0.84698784
CHEMBL3596868	0.8478155	0.8470007	0.84703493
CHEMBL66612	0.84769285	0.8467978	0.8467511
CHEMBL1761943	0.8475003	0.84677577	0.8464964
CHEMBL94487	0.8474416	0.84709	0.8471117
CHEMBL524111	0.84741634	0.84721386	0.8471946
CHEMBL1081138	0.8471767	0.84707725	0.84675664
CHEMBL3641196	0.84715575	0.84568626	0.84613144
CHEMBL3929448	0.84667563	0.84592116	0.84587336
CHEMBL1761944	0.8465892	0.846095	0.845959
CHEMBL1171836	0.8464526	0.8459747	0.8460167
CHEMBL287852	0.84626436	0.8454191	0.84494275
CHEMBL1079354	0.8462284	0.8458011	0.8457246
CHEMBL3641105	0.8461789	0.84498477	0.8448894
CHEMBL1081169	0.8459435	0.8454208	0.8453671
CHEMBL525487	0.84580415	0.84520966	0.8450672

CHEMBL3657241	0.845755	0.8453937	0.84548104
CHEMBL3604939	0.84573996	0.8452582	0.8451324
CHEMBL3596889	0.84572583	0.84474087	0.844579
CHEMBL520742	0.845642	0.8450005	0.8442645
CHEMBL3739427	0.84559643	0.8452448	0.84505486
CHEMBL4065878	0.84543574	0.8445741	0.84426177
CHEMBL1824069	0.84540474	0.844655	0.8448519
CHEMBL1824291	0.84539115	0.84461063	0.84477174
CHEMBL1824297	0.84539115	0.84461063	0.84477174
CHEMBL420885	0.84538054	0.84469867	0.8441926
CHEMBL345645	0.84527254	0.844928	0.8445046
CHEMBL4069942	0.8451066	0.84484303	0.84428537
CHEMBL404571	0.8450976	0.84469867	0.8442229
CHEMBL167890	0.84508777	0.8446482	0.84455585
CHEMBL1761933	0.84499854	0.84471905	0.84450203
CHEMBL30359	0.84499115	0.8443327	0.84406006
CHEMBL378687	0.8447532	0.8439215	0.8436998
CHEMBL67436	0.8446637	0.8441651	0.8444222
CHEMBL2024461	0.84423673	0.8433069	0.8429326
CHEMBL3133752	0.8439814	0.8434949	0.8431648
CHEMBL3659909	0.84374845	0.84283125	0.84284246
CHEMBL530760	0.8436121	0.8431658	0.8426997
CHEMBL169725	0.8435566	0.84291404	0.84265137
CHEMBL2089215	0.84342873	0.8431755	0.84337795
CHEMBL104206	0.8429779	0.8423253	0.8424964
CHEMBL2113176	0.84278214	0.8422636	0.84197205
CHEMBL3957025	0.84267974	0.84205973	0.84204805
CHEMBL2203438	0.842361	0.84191716	0.8416637
CHEMBL3646738	0.8421082	0.84158427	0.8415304
CHEMBL3732259	0.84198916	0.84161484	0.8412733
CHEMBL3727757	0.84158766	0.84147674	0.8410255
CHEMBL4172791	0.8414911	0.8408412	0.8407396
CHEMBL121829	0.8414549	0.8413829	0.8410425
CHEMBL527153	0.8413137	0.8411015	0.8406539
CHEMBL3740113	0.84131193	0.8410643	0.840721
CHEMBL2029987	0.84126186	0.84054726	0.8405293
CHEMBL3740418	0.8411652	0.840593	0.8401045
CHEMBL206374	0.8410425	0.84055495	0.8406114
CHEMBL3646706	0.84100604	0.8405144	0.8403616
CHEMBL593292	0.8409016	0.8404888	0.84024227
CHEMBL595615	0.8409016	0.8404888	0.84024227
CHEMBL4065359	0.84089816	0.8402647	0.8402204
CHEMBL2335868	0.8408244	0.84063506	0.8401681
CHEMBL3716588	0.840578	0.8402027	0.8399451
CHEMBL4160840	0.8405396	0.8401456	0.8400171
CHEMBL514343	0.8405057	0.84008586	0.8402995
CHEMBL58652	0.8403511	0.83955973	0.83886456
CHEMBL1643983	0.8401401	0.83930266	0.8387317
CHEMBL3700343	0.8401391	0.8395671	0.8393123
CHEMBL1760036	0.8399658	0.8395883	0.8395326

CHEMBL3604922	0.8398869	0.8392414	0.8387965
CHEMBL3731216	0.83986735	0.83923984	0.83891743
CHEMBL272524	0.8397625	0.84001607	0.8403424
CHEMBL2386793	0.8397453	0.8391868	0.8390806
CHEMBL2335878	0.8395062	0.83924615	0.8386998
CHEMBL165265	0.8394606	0.83900034	0.8391824
CHEMBL3601705	0.8394176	0.83890826	0.83842397
CHEMBL1824299	0.83926624	0.8385114	0.83877414
CHEMBL1089141	0.8392068	0.838762	0.8384434
CHEMBL1824304	0.8390833	0.83828807	0.838523
CHEMBL2018764	0.8390311	0.83850276	0.83824223
CHEMBL3618131	0.83887446	0.83844864	0.83849704
CHEMBL3729123	0.8388566	0.83857226	0.838293
CHEMBL3731098	0.8387719	0.8384584	0.8382615
CHEMBL1824300	0.838725	0.8377819	0.8380319
CHEMBL3694125	0.83868945	0.83827496	0.83831924
CHEMBL3601226	0.8385569	0.8375019	0.83690274
CHEMBL3983460	0.83842546	0.8380398	0.83772624
CHEMBL3731375	0.838269	0.8376348	0.8373646
CHEMBL3732318	0.8382647	0.83795464	0.8377405
CHEMBL1824290	0.83825463	0.83756995	0.8376374
CHEMBL1824296	0.83825463	0.83756995	0.8376374
CHEMBL547668	0.8382304	0.8381569	0.83767915
CHEMBL489913	0.8381646	0.836975	0.8366858
CHEMBL4084436	0.83804023	0.837664	0.83744025
CHEMBL166031	0.8380224	0.8376453	0.8377009
CHEMBL3593319	0.83800566	0.83741736	0.83722454
CHEMBL3628796	0.8379431	0.83739007	0.837114
CHEMBL2335882	0.83770186	0.83752704	0.837121
CHEMBL3787662	0.8375108	0.83685434	0.8363186
CHEMBL2386794	0.8374332	0.83691794	0.8367763
CHEMBL4099854	0.83723414	0.83651984	0.8362473
CHEMBL489058	0.8371781	0.8367624	0.83615744
CHEMBL1078027	0.8371289	0.83648574	0.83633375
CHEMBL2386816	0.83707863	0.83669186	0.8364067
CHEMBL2336027	0.83705705	0.8358473	0.83543175
CHEMBL4095503	0.83699286	0.83632714	0.8356199
CHEMBL30540	0.83693546	0.8364095	0.83598626
CHEMBL444829	0.83686376	0.8358819	0.8352517
CHEMBL3968039	0.83660823	0.83558017	0.8354273
CHEMBL534617	0.8365196	0.8361506	0.8357372
CHEMBL3742260	0.8365123	0.83575886	0.83550954
CHEMBL3740327	0.83635634	0.83563733	0.83519316
CHEMBL3740144	0.8361986	0.8358209	0.8354452
CHEMBL3116229	0.8361962	0.8364289	0.83588666
CHEMBL3799035	0.83612245	0.8357229	0.8354753
CHEMBL1165535	0.8361212	0.83589137	0.8358003
CHEMBL3759754	0.8361085	0.8354323	0.83518636
CHEMBL4277564	0.8361018	0.8360976	0.8359461
CHEMBL1213922	0.83608305	0.8356362	0.83537054

CHEMBL1080064	0.8360349	0.83542687	0.83539015
CHEMBL1795970	0.8359594	0.8358916	0.83552307
CHEMBL3393606	0.83584875	0.8355803	0.83530855
CHEMBL1643986	0.8358072	0.834551	0.8339038
CHEMBL4166026	0.8356995	0.8348998	0.83486676
CHEMBL30763	0.83550537	0.8346534	0.83424324
CHEMBL256527	0.8354673	0.83490336	0.8343091
CHEMBL214214	0.8354624	0.83421546	0.83368737
CHEMBL4105091	0.8353471	0.83513737	0.835161
CHEMBL106068	0.8351211	0.8345815	0.83470887
CHEMBL1914662	0.8350502	0.8346516	0.8344257
CHEMBL3099974	0.8350037	0.83479583	0.8342107
CHEMBL3739946	0.83494735	0.8345069	0.83399695
CHEMBL3727498	0.83488995	0.83432615	0.8341996
CHEMBL4098951	0.83471763	0.8340694	0.8334596
CHEMBL79560	0.83466715	0.8346345	0.83453596
CHEMBL3133754	0.83466256	0.8339843	0.83359313
CHEMBL285956	0.8345988	0.8338731	0.83337754
CHEMBL1196064	0.834553	0.83426154	0.8340852
CHEMBL71191	0.834486	0.8343407	0.83432114
CHEMBL3542333	0.834478	0.83423865	0.83364004
CHEMBL3641136	0.8344762	0.83400714	0.83364576
CHEMBL1824287	0.83447206	0.834054	0.8339795
CHEMBL300791	0.83438575	0.83390105	0.8338482
CHEMBL148259	0.83436686	0.834156	0.83412176
CHEMBL448729	0.83436686	0.834156	0.83412176
CHEMBL1257807	0.83430743	0.83415496	0.83374286
CHEMBL90541	0.8342725	0.8339156	0.8337462
CHEMBL99024	0.83426	0.832743	0.8325216
CHEMBL4169668	0.834218	0.8334246	0.8330839
CHEMBL13976	0.8341011	0.83372504	0.8332639
CHEMBL3809588	0.83405215	0.83365905	0.8338245
CHEMBL60979	0.8338899	0.8328775	0.8323198
CHEMBL4283353	0.8338883	0.8340305	0.8336361
CHEMBL2207759	0.83381	0.83260125	0.8323295
CHEMBL1082075	0.83364797	0.833045	0.8329766
CHEMBL186851	0.83363926	0.8332473	0.83326364
CHEMBL4066470	0.83326846	0.8324716	0.83181995
CHEMBL3731622	0.83323646	0.83274984	0.8326395
CHEMBL1963468	0.83321095	0.83282673	0.8325795
CHEMBL3780571	0.83315945	0.8335392	0.8329642
CHEMBL3646709	0.8331241	0.8324232	0.83240134
CHEMBL185921	0.8328515	0.8323324	0.83244044
CHEMBL1094704	0.832703	0.83226526	0.8320623
CHEMBL3909969	0.8326303	0.8321364	0.83208025
CHEMBL63115	0.832516	0.83183444	0.8317564
CHEMBL526321	0.83244216	0.8311341	0.8310994
CHEMBL3596862	0.8324189	0.8315676	0.83154726
CHEMBL3593673	0.83216894	0.8315799	0.8315567
CHEMBL3694137	0.83212566	0.83121014	0.8312025

CHEMBL67424	0.83206296	0.8313262	0.8314541
CHEMBL184575	0.83194447	0.83121777	0.8310124
CHEMBL489914	0.8318584	0.83083624	0.8305626
CHEMBL2018749	0.8318374	0.831587	0.831324
CHEMBL132466	0.83164847	0.8302918	0.8299266
CHEMBL547186	0.83163196	0.83132344	0.83113164
CHEMBL107901	0.8316038	0.8311106	0.8313179
CHEMBL491574	0.83152103	0.8305468	0.8303826
CHEMBL2029978	0.8314887	0.8307422	0.83081585
CHEMBL4095291	0.8313936	0.83118296	0.8313437
CHEMBL1990885	0.83135295	0.8309217	0.83072686
CHEMBL3410031	0.83122635	0.830429	0.8299279
CHEMBL1761934	0.8311244	0.8307364	0.83054405
CHEMBL432396	0.8310483	0.8297152	0.82918483
CHEMBL3604935	0.83097136	0.83048785	0.8301564
CHEMBL202241	0.8309198	0.8299599	0.8298751
CHEMBL379001	0.8307759	0.8304174	0.8302883
CHEMBL491677	0.8307081	0.83056056	0.83033574
CHEMBL1080784	0.83069944	0.8302107	0.83016604
CHEMBL236487	0.8306736	0.83021134	0.82999504
CHEMBL1080783	0.83054173	0.829891	0.829719
CHEMBL3133753	0.83049476	0.82990944	0.82972
CHEMBL3942536	0.83040726	0.8299992	0.8296467
CHEMBL4175071	0.83029294	0.8305468	0.83006227
CHEMBL3426224	0.8302642	0.82950026	0.82904816
CHEMBL102346	0.8301072	0.82981414	0.8300289
CHEMBL3951268	0.83009326	0.8294074	0.82900155
CHEMBL1213975	0.8300655	0.82979596	0.82947046
CHEMBL179633	0.83000237	0.8293742	0.8290085
CHEMBL593860	0.8299831	0.8295493	0.8294103
CHEMBL593911	0.8299831	0.8295493	0.8294103
CHEMBL125898	0.8296894	0.82918906	0.8293322
CHEMBL3716079	0.8295634	0.829265	0.82917684
CHEMBL2417991	0.829546	0.82932305	0.8289095
CHEMBL204217	0.82952464	0.8286804	0.8283209
CHEMBL3924442	0.82952327	0.82942694	0.82909644
CHEMBL270348	0.82949775	0.82921636	0.8287201
CHEMBL473761	0.8294753	0.8293213	0.8287066
CHEMBL536206	0.82940936	0.82922393	0.82893807
CHEMBL3758749	0.82927895	0.82871455	0.8284451
CHEMBL1807196	0.82925874	0.8289053	0.8288975
CHEMBL4175773	0.8289572	0.82829607	0.8282125
CHEMBL105032	0.8288896	0.8284377	0.82851887
CHEMBL31733	0.8288241	0.82825863	0.82788706
CHEMBL3099962	0.8287205	0.82873344	0.82822627
CHEMBL3727485	0.82865775	0.82815045	0.8280158
CHEMBL3727716	0.82859296	0.8281862	0.82806784
CHEMBL517069	0.8285187	0.8277886	0.8274001
CHEMBL2386789	0.82838726	0.827687	0.8275939
CHEMBL166030	0.828303	0.8279103	0.82799584

CHEMBL3957061	0.8282398	0.8276131	0.8276006
CHEMBL1203938	0.82823366	0.82777846	0.8275746
CHEMBL1079545	0.8281817	0.8274941	0.82737505
CHEMBL3933670	0.8281493	0.8279142	0.82737684
CHEMBL1270279	0.8280636	0.8275218	0.82761526
CHEMBL4171831	0.8279458	0.8276225	0.8277177
CHEMBL349575	0.82790995	0.82762533	0.8271611
CHEMBL4228018	0.82789016	0.8276371	0.82720476
CHEMBL3729382	0.82781935	0.82738	0.82718134
CHEMBL3040817	0.8278036	0.82757914	0.827191
CHEMBL248674	0.82751375	0.82688487	0.82685673
CHEMBL1643976	0.82741463	0.8259423	0.82534254
CHEMBL490711	0.8273835	0.8265714	0.82634926
CHEMBL1761931	0.82737935	0.826983	0.8267938
CHEMBL4086642	0.82734376	0.82685876	0.82670164
CHEMBL184382	0.82721376	0.8265014	0.82633996
CHEMBL215191	0.827126	0.8269827	0.826663
CHEMBL4282624	0.8271129	0.8273281	0.82691115
CHEMBL4287582	0.8271129	0.8273281	0.82691115
CHEMBL4294819	0.8271129	0.8273281	0.82691115
CHEMBL3932057	0.8271023	0.82685566	0.8267074
CHEMBL382667	0.82707095	0.82625294	0.8261018
CHEMBL485337	0.82703507	0.8268504	0.8266119
CHEMBL45683	0.827009	0.82684	0.82655513
CHEMBL1795965	0.8269909	0.8267977	0.8264836
CHEMBL3809715	0.8269602	0.8263833	0.8262285
CHEMBL31276	0.8267613	0.82611156	0.82557493
CHEMBL481727	0.82675624	0.82588005	0.82551056
CHEMBL3730376	0.82673806	0.82626426	0.8261436
CHEMBL4213724	0.8266159	0.82653904	0.8261431
CHEMBL3646701	0.8265593	0.8263285	0.82627624
CHEMBL4071399	0.82643574	0.8262822	0.8260703
CHEMBL556669	0.8263976	0.8256737	0.82562935
CHEMBL2203431	0.82638454	0.82606244	0.8259661
CHEMBL490944	0.82636726	0.8254505	0.8251735
CHEMBL124313	0.82636094	0.8260464	0.82630616
CHEMBL3938494	0.82629174	0.8251625	0.8248578
CHEMBL96065	0.8261024	0.82567126	0.82563233
CHEMBL3739992	0.82609093	0.8255738	0.82555544
CHEMBL3697861	0.8260628	0.8251098	0.82498276
CHEMBL523713	0.8259004	0.82502276	0.82462156
CHEMBL29641	0.8258741	0.8249669	0.82484716
CHEMBL4211604	0.8257811	0.8246592	0.8241786
CHEMBL3694119	0.8256763	0.82501495	0.8253647
CHEMBL3237184	0.82566553	0.82530147	0.8251411
CHEMBL575307	0.82564783	0.82512563	0.82507885
CHEMBL3604929	0.8254863	0.82469565	0.82433945
CHEMBL1256429	0.8254693	0.8249817	0.82457405
CHEMBL3597572	0.825328	0.8250619	0.82438105
CHEMBL1824072	0.82529944	0.8242654	0.82397115

CHEMBL1095498	0.82528293	0.8251718	0.8243947
CHEMBL2436956	0.824939	0.8248925	0.82439953
CHEMBL3601231	0.8249086	0.82418	0.82368636
CHEMBL3965994	0.82483864	0.8244803	0.82409465
CHEMBL219722	0.8248008	0.82495207	0.8247609
CHEMBL584293	0.8247764	0.82437605	0.8243266
CHEMBL3890300	0.8247061	0.82456934	0.8241035
CHEMBL4286870	0.82454014	0.82382244	0.8234942
CHEMBL1078453	0.82453203	0.82368594	0.82359976
CHEMBL222728	0.8244773	0.8243527	0.8240317
CHEMBL1774649	0.82436407	0.82403994	0.8238723
CHEMBL224013	0.82436407	0.82403994	0.8238723
CHEMBL91428	0.8242672	0.82375246	0.8236178
CHEMBL124895	0.82423735	0.82384515	0.82396007
CHEMBL1795959	0.8241738	0.8239249	0.8234979
CHEMBL1939659	0.8240754	0.8226824	0.822446
CHEMBL3798940	0.8239598	0.8236063	0.8235434
CHEMBL4291935	0.8238565	0.82328105	0.8228942
CHEMBL2029972	0.8238518	0.8233179	0.82325757
CHEMBL2029990	0.8237911	0.8229389	0.8228302
CHEMBL1985006	0.82361996	0.82301104	0.8223804
CHEMBL518981	0.82347906	0.82283556	0.8226994
CHEMBL3728101	0.82343817	0.8231407	0.82292306
CHEMBL575925	0.8234148	0.8227378	0.82250845
CHEMBL3909341	0.8234071	0.8229398	0.8228287
CHEMBL4170459	0.82325846	0.82261086	0.82236344
CHEMBL3040795	0.8232431	0.82301223	0.82267666
CHEMBL3962948	0.82312995	0.82243776	0.82242155
CHEMBL2335890	0.8230691	0.8228523	0.8225113
CHEMBL4075068	0.82305515	0.82229483	0.8219269
CHEMBL3421636	0.82304466	0.8235665	0.8235637
CHEMBL183929	0.8229015	0.8221492	0.82200927
CHEMBL4083001	0.8227984	0.8223033	0.8219072
CHEMBL3410032	0.82278985	0.8220438	0.82160306
CHEMBL3758875	0.82276976	0.8221247	0.8218653
CHEMBL1256421	0.8226731	0.8223154	0.8220974
CHEMBL1256432	0.8226731	0.8223154	0.8220974
CHEMBL2203432	0.8226008	0.8220458	0.8217468
CHEMBL3740588	0.82248074	0.8220009	0.8219799
CHEMBL30728	0.8224675	0.8216709	0.821173
CHEMBL309682	0.822461	0.8221777	0.8222027
CHEMBL452716	0.82239294	0.8217089	0.82124233
CHEMBL4171747	0.8223617	0.82164156	0.8214736
CHEMBL3641202	0.8222186	0.82112265	0.8213636
CHEMBL2018772	0.8219124	0.82122517	0.8209269
CHEMBL3685423	0.82174945	0.82136595	0.8207711
CHEMBL571434	0.8217182	0.8207239	0.820599
CHEMBL4102688	0.82166743	0.82101625	0.8208245
CHEMBL4225571	0.8215821	0.82147527	0.8210907
CHEMBL3343038	0.8213999	0.8209689	0.82069284

CHEMBL3265000	0.8213203	0.82084084	0.82072157
CHEMBL2018748	0.8212909	0.8210454	0.8208306
CHEMBL3809895	0.8212588	0.8210262	0.8210803
CHEMBL46785	0.82110125	0.8209537	0.8208
CHEMBL2208033	0.821023	0.8205962	0.82043695
CHEMBL3953109	0.8207084	0.8204111	0.82037055
CHEMBL102427	0.82066035	0.8203535	0.82052374
CHEMBL4228493	0.82065564	0.8202209	0.81960756
CHEMBL3628253	0.8206183	0.8206912	0.8203312
CHEMBL593306	0.820604	0.82037675	0.8202115
CHEMBL574039	0.8205955	0.8202086	0.8201151
CHEMBL1795962	0.8204752	0.8201596	0.8199763
CHEMBL30516	0.82037306	0.81916714	0.81874895
CHEMBL109372	0.82029015	0.8190054	0.81874925
CHEMBL1276892	0.820264	0.8196583	0.82007295
CHEMBL582415	0.8202343	0.8200705	0.81968975
CHEMBL4099804	0.8202322	0.82004774	0.8201821
CHEMBL2203437	0.8201762	0.81931216	0.8188561
CHEMBL31384	0.8201623	0.81918824	0.81882894
CHEMBL1761937	0.81994265	0.819629	0.81955874
CHEMBL84179	0.8199301	0.8184777	0.8179146
CHEMBL2018882	0.81992114	0.81945884	0.81929135
CHEMBL1277254	0.8199145	0.8193276	0.81936485
CHEMBL239160	0.8199109	0.8193227	0.81890404
CHEMBL81073	0.81986284	0.81888723	0.8185721
CHEMBL3694156	0.81984043	0.81889087	0.8186517
CHEMBL2417874	0.81962764	0.81928605	0.8189538
CHEMBL321078	0.8196144	0.8182572	0.81800926
CHEMBL417740	0.81941414	0.81848353	0.81838834
CHEMBL4162113	0.81937194	0.81845623	0.8183147
CHEMBL257454	0.81937146	0.8191588	0.8194761
CHEMBL186802	0.8193597	0.8188536	0.81856126
CHEMBL263001	0.81932795	0.81899726	0.8185556
CHEMBL3732687	0.8193079	0.81884503	0.81877863
CHEMBL3355048	0.8192972	0.81876403	0.8184638
CHEMBL3589745	0.8192739	0.81919837	0.81881887
CHEMBL1739092	0.8192034	0.8187806	0.81844926
CHEMBL4085962	0.8191552	0.8190491	0.8186407
CHEMBL70130	0.81913376	0.8184551	0.8182261
CHEMBL223304	0.8189801	0.8187757	0.81852424
CHEMBL3329397	0.818938	0.81828856	0.8180069
CHEMBL2333992	0.8189243	0.8187429	0.81862867
CHEMBL3758796	0.8188633	0.8183129	0.81801885
CHEMBL202567	0.8187285	0.8180022	0.81770873
CHEMBL1242116	0.8185772	0.8182143	0.81803447
CHEMBL3650740	0.81850785	0.81834394	0.8179156
CHEMBL4215501	0.8184848	0.81773305	0.8173411
CHEMBL3740115	0.81832117	0.8176976	0.8174179
CHEMBL162034	0.8180723	0.8181004	0.81773967
CHEMBL3759155	0.818039	0.8175751	0.8172666

CHEMBL3265001	0.8179184	0.8176384	0.8175032
CHEMBL1761929	0.81791544	0.8174758	0.81723166
CHEMBL3741008	0.81791246	0.817018	0.8163386
CHEMBL1807198	0.8179121	0.8179358	0.8176669
CHEMBL4171543	0.81780195	0.8175415	0.81723744
CHEMBL1455018	0.81778055	0.81753266	0.8173466
CHEMBL2136735	0.81774807	0.8173186	0.8170203
CHEMBL2113170	0.81771404	0.8173489	0.8168061
CHEMBL4070814	0.8176538	0.8170702	0.8166212
CHEMBL353584	0.8176266	0.8172694	0.8173488
CHEMBL318326	0.81758493	0.8161305	0.8157627
CHEMBL426004	0.8175202	0.8170451	0.81676066
CHEMBL418111	0.81744945	0.81676495	0.8161653
CHEMBL324576	0.81744695	0.81658673	0.816572
CHEMBL514505	0.8174367	0.8167009	0.81722265
CHEMBL2047988	0.81738055	0.817288	0.8175195
CHEMBL1802771	0.8173503	0.8167211	0.8169231
CHEMBL529926	0.81733155	0.8170426	0.81669843
CHEMBL202502	0.8172987	0.81690615	0.81674916
CHEMBL3740535	0.81724393	0.81700116	0.8168223
CHEMBL3728775	0.8172374	0.8170629	0.81686735
CHEMBL179577	0.81721765	0.8166966	0.8162986
CHEMBL1163731	0.81719106	0.81624514	0.8157958
CHEMBL1163979	0.81719106	0.81624514	0.8157958
CHEMBL575905	0.81718856	0.8168404	0.81707704
CHEMBL66251	0.817097	0.81693155	0.8168374
CHEMBL66329	0.81705314	0.8166747	0.81643987
CHEMBL586700	0.8170444	0.8166472	0.81649303
CHEMBL403732	0.81700957	0.8166553	0.8166325
CHEMBL419428	0.8169569	0.8161432	0.8157456
CHEMBL1369868	0.8168503	0.8163254	0.81614256
CHEMBL531673	0.81679624	0.81675524	0.816265
CHEMBL3799354	0.81679183	0.8165578	0.8166588
CHEMBL4075453	0.8167774	0.81643367	0.8162541
CHEMBL1885536	0.8167181	0.8160998	0.816118
CHEMBL531447	0.8166552	0.8162087	0.81625247
CHEMBL1824289	0.8165522	0.81601024	0.8162297
CHEMBL1824295	0.8165522	0.81601024	0.8162297
CHEMBL3099973	0.81644666	0.8164419	0.8158264
CHEMBL574349	0.81642354	0.8160132	0.81591403
CHEMBL4210398	0.81638575	0.8159949	0.8159114
CHEMBL4214803	0.81638575	0.8159949	0.8159114
CHEMBL3288850	0.8163539	0.8160381	0.81589913
CHEMBL3321909	0.8163159	0.8159532	0.8159775
CHEMBL3659917	0.81629544	0.8150842	0.81537545
CHEMBL202721	0.81629074	0.815797	0.8155083
CHEMBL3956409	0.81625324	0.8158146	0.81535715
CHEMBL56142	0.81616896	0.81558514	0.8155217
CHEMBL1081345	0.81616354	0.8153634	0.815362
CHEMBL3237185	0.8161212	0.8160193	0.8158499

CHEMBL522791	0.81603175	0.8149209	0.81457543
CHEMBL2141478	0.81602705	0.8156319	0.815433
CHEMBL484270	0.81602705	0.8156319	0.815433
CHEMBL247790	0.8159995	0.8157483	0.81563324
CHEMBL1744086	0.8159908	0.815401	0.815482
CHEMBL1788321	0.8159908	0.815401	0.815482
CHEMBL2022966	0.81579244	0.8152963	0.8153105
CHEMBL312385	0.8157344	0.8141979	0.81367135
CHEMBL2022964	0.81546557	0.8145988	0.81454533
CHEMBL3758355	0.8152908	0.8148194	0.81448233
CHEMBL424943	0.8151691	0.8148016	0.8143677
CHEMBL3809608	0.8151642	0.8143792	0.814389
CHEMBL4276786	0.8151588	0.81452096	0.8142128
CHEMBL178096	0.8151245	0.8146466	0.81444347
CHEMBL3977504	0.81510437	0.8148736	0.8146584
CHEMBL4162529	0.81510305	0.8142574	0.8141338
CHEMBL3604942	0.81510043	0.81475616	0.8147153
CHEMBL4080534	0.81508994	0.8147705	0.81495285
CHEMBL4226965	0.81500447	0.81464356	0.8143033
CHEMBL3977662	0.8149884	0.81425524	0.81407505
CHEMBL1094706	0.814922	0.8146356	0.8144698
CHEMBL3651204	0.81479555	0.8144312	0.8139069
CHEMBL3916001	0.814775	0.8143714	0.814211
CHEMBL65850	0.814775	0.8147107	0.81422704
CHEMBL3989134	0.8146508	0.81442964	0.81416726
CHEMBL3740228	0.8145469	0.81410545	0.81363916
CHEMBL4080720	0.81450605	0.8141529	0.8140901
CHEMBL386310	0.8144918	0.81399965	0.8141511
CHEMBL168885	0.8143656	0.8139503	0.8136682
CHEMBL4170111	0.8143264	0.8135947	0.8132488
CHEMBL1257445	0.8143141	0.8135542	0.8131354
CHEMBL4170648	0.81421053	0.8138465	0.81380856
CHEMBL555844	0.81418633	0.8138659	0.8137491
CHEMBL194882	0.8141546	0.81418407	0.8140328
CHEMBL4169209	0.8140757	0.81339616	0.8131428
CHEMBL3740578	0.81401896	0.8138083	0.8135913
CHEMBL1079544	0.8139631	0.8135625	0.81343555
CHEMBL3739890	0.81378573	0.8132409	0.8128929
CHEMBL1761939	0.81377506	0.8134799	0.8134518
CHEMBL340200	0.8136843	0.81322503	0.8132345
CHEMBL3758363	0.81364	0.8131423	0.8127323
CHEMBL3593289	0.8136288	0.812369	0.8119734
CHEMBL471520	0.8136195	0.81336355	0.8130469
CHEMBL1236904	0.81359285	0.81314534	0.8127262
CHEMBL212762	0.8135617	0.8132282	0.8126316
CHEMBL3894623	0.8135483	0.8132037	0.8127177
CHEMBL3741130	0.81349427	0.8128645	0.8124774
CHEMBL57758	0.8134867	0.8129395	0.8128954
CHEMBL3633292	0.81339204	0.81327355	0.81293464
CHEMBL3735656	0.81336105	0.8126444	0.81234753

CHEMBL3593314	0.81335163	0.8125184	0.81251156
CHEMBL1824075	0.8133398	0.8127293	0.8127684
CHEMBL535038	0.8132975	0.8131921	0.81329155
CHEMBL3824185	0.81323034	0.81151277	0.8109721
CHEMBL3962740	0.8132001	0.8125718	0.81204134
CHEMBL375236	0.8131867	0.813293	0.8129436
CHEMBL467629	0.8131416	0.812895	0.81280315
CHEMBL3728889	0.8131336	0.81263876	0.81250274
CHEMBL359830	0.81307507	0.8126467	0.81236935
CHEMBL3918261	0.8130119	0.81256545	0.8123787
CHEMBL184699	0.81298244	0.81277347	0.8127546
CHEMBL437197	0.8129643	0.8125721	0.81247795
CHEMBL4172592	0.81283236	0.81229544	0.8122537
CHEMBL3741325	0.8127568	0.8120402	0.8115862
CHEMBL1078689	0.81273186	0.81265396	0.8126923
CHEMBL1974582	0.8124824	0.81177187	0.8120556
CHEMBL1278235	0.8124619	0.8121978	0.81230426
CHEMBL2203433	0.81243455	0.81229395	0.8122026
CHEMBL3655750	0.8123457	0.81206334	0.8122844
CHEMBL3655752	0.8123457	0.81206334	0.8122844
CHEMBL179224	0.81231517	0.81190646	0.81161416
CHEMBL3039689	0.8123004	0.81214195	0.8117042
CHEMBL54067	0.8122653	0.8117656	0.81129324
CHEMBL166255	0.8122149	0.81205845	0.811731
CHEMBL337728	0.8121374	0.81140566	0.81084657
CHEMBL3954794	0.8121264	0.81158257	0.8115673
CHEMBL3904787	0.8121127	0.811497	0.8111507
CHEMBL2333991	0.81205606	0.81169826	0.8115436
CHEMBL588434	0.8120287	0.8114622	0.8111286
CHEMBL3265023	0.81199634	0.8122138	0.8119442
CHEMBL214601	0.8119553	0.8115586	0.8115337
CHEMBL3759724	0.81193465	0.81144905	0.81110644
CHEMBL1683651	0.8119191	0.8113816	0.8112161
CHEMBL3589807	0.8119148	0.8117252	0.8113719
CHEMBL1643996	0.81188464	0.81093615	0.8102341
CHEMBL1080411	0.81184363	0.81137663	0.81098366
CHEMBL4203738	0.8118139	0.8114165	0.81098545
CHEMBL3930890	0.8117032	0.8106725	0.8108916
CHEMBL3964955	0.81168354	0.8115499	0.81152236
CHEMBL1171283	0.81161505	0.81092256	0.8105415
CHEMBL325245	0.81139064	0.81119925	0.8109918
CHEMBL164272	0.8113525	0.81094825	0.8106481
CHEMBL420672	0.81128436	0.8101906	0.8102526
CHEMBL180331	0.8112359	0.8107948	0.8108541
CHEMBL66932	0.8111359	0.8105189	0.81006706
CHEMBL2333993	0.811127	0.81091446	0.8108295
CHEMBL14699	0.8110959	0.8106009	0.81057686
CHEMBL215028	0.8110869	0.8109377	0.8112348
CHEMBL3740310	0.81108457	0.8105904	0.8106109
CHEMBL4067978	0.81096494	0.8095274	0.8093548

CHEMBL1761945	0.8108684	0.81041217	0.8104239
CHEMBL573538	0.8108215	0.8101716	0.80980897
CHEMBL165283	0.8108035	0.8105688	0.8102536
CHEMBL529243	0.81079996	0.8103911	0.81012774
CHEMBL479846	0.8107749	0.8097396	0.80905986
CHEMBL4165965	0.81073016	0.8100478	0.8099201
CHEMBL30551	0.81066316	0.8096418	0.80917746
CHEMBL1824074	0.8106508	0.81006074	0.8102145
CHEMBL2029973	0.8106122	0.81011796	0.8101163
CHEMBL223185	0.8105611	0.8104461	0.8101056
CHEMBL1808993	0.8104375	0.810041	0.80981547
CHEMBL451163	0.8103368	0.8099224	0.8097881
CHEMBL4100426	0.81027853	0.8097755	0.8097807
CHEMBL548789	0.81021476	0.8100455	0.8097428
CHEMBL3917723	0.81021124	0.8094913	0.8089491
CHEMBL4062758	0.81004435	0.80966485	0.809358
CHEMBL2336011	0.8098352	0.80890286	0.8084867
CHEMBL292785	0.8098296	0.8093829	0.80942565
CHEMBL3920551	0.80977225	0.80877745	0.8086346
CHEMBL1761940	0.8096963	0.8093411	0.8091712
CHEMBL4100979	0.80954015	0.8087758	0.80841744
CHEMBL144990	0.809523	0.8092429	0.80920154
CHEMBL3758900	0.80945516	0.80898684	0.8086826
CHEMBL203507	0.80939186	0.80867636	0.8084934
CHEMBL3403671	0.8093749	0.80898213	0.80870354
CHEMBL2029970	0.80935895	0.8087548	0.8089453
CHEMBL1271393	0.80934596	0.8088363	0.8087925
CHEMBL125431	0.8093456	0.808974	0.80900323
CHEMBL4082210	0.80932546	0.80829394	0.80740774
CHEMBL1807781	0.8093146	0.8089218	0.80891335
CHEMBL3798986	0.80930734	0.8093488	0.8092942
CHEMBL1164935	0.8093058	0.8095012	0.8092344
CHEMBL2335874	0.8092295	0.8092307	0.8087906
CHEMBL118130	0.8091719	0.8084798	0.8082091
CHEMBL4279865	0.80916303	0.80936265	0.8089256
CHEMBL3740224	0.80909115	0.80863845	0.8087588
CHEMBL481231	0.8090632	0.8083185	0.8080553
CHEMBL481402	0.8090632	0.8083185	0.8080553
CHEMBL1999749	0.8089665	0.8084308	0.80822307
CHEMBL3890526	0.8089665	0.8084308	0.80822307
CHEMBL458098	0.8089665	0.8084308	0.80822307
CHEMBL581604	0.80890554	0.8088727	0.80856365
CHEMBL3542323	0.8087913	0.8086451	0.8087707
CHEMBL3221551	0.80870426	0.8084291	0.808241
CHEMBL1824303	0.8086796	0.80814075	0.80842495
CHEMBL1824306	0.8086796	0.80814075	0.80842495
CHEMBL144725	0.8085005	0.8079865	0.80819684
CHEMBL339685	0.8085005	0.8079865	0.80819684
CHEMBL3393607	0.80846393	0.8081761	0.807902
CHEMBL304971	0.80843997	0.8082343	0.8078656

CHEMBL582000	0.8084359	0.80878884	0.808855
CHEMBL3976245	0.80840826	0.808277	0.808018
CHEMBL303704	0.8083599	0.8076555	0.8072467
CHEMBL593760	0.80832696	0.8077888	0.80769986
CHEMBL593987	0.80832696	0.8077888	0.80769986
CHEMBL3716854	0.8082971	0.80820024	0.8079258
CHEMBL3650744	0.80827594	0.80799437	0.80768734
CHEMBL178306	0.80825114	0.8074956	0.8070804
CHEMBL3633684	0.80824566	0.80787617	0.8075454
CHEMBL81712	0.808243	0.8075665	0.8071901
CHEMBL533085	0.8082348	0.8072884	0.8071573
CHEMBL3265020	0.8081821	0.80806065	0.8075514
CHEMBL2042953	0.80816424	0.80796	0.80796117
CHEMBL256529	0.8080974	0.8073871	0.80712855
CHEMBL1221767	0.80801564	0.8075899	0.80745304
CHEMBL4159888	0.8079585	0.80717176	0.8069023
CHEMBL4166390	0.8078865	0.80766785	0.80783856
CHEMBL4169642	0.80786574	0.80755734	0.8075514
CHEMBL3739615	0.80774975	0.80701554	0.80656224
CHEMBL2029981	0.8076728	0.8067725	0.806646
CHEMBL124439	0.80763316	0.80725795	0.80720854
CHEMBL3601722	0.8074625	0.8071841	0.80677825
CHEMBL2203428	0.8074346	0.8069614	0.8067663
CHEMBL1256424	0.8073914	0.80706704	0.806653
CHEMBL1774648	0.80733263	0.8069784	0.80685985
CHEMBL94431	0.80733263	0.8069784	0.80685985
CHEMBL2089212	0.807243	0.80702984	0.8071596
CHEMBL4173072	0.8072162	0.8066985	0.80617094
CHEMBL4284071	0.8071995	0.80631804	0.80579114
CHEMBL535947	0.8071172	0.80652106	0.80644065
CHEMBL1824073	0.8069957	0.8058592	0.80578125
CHEMBL1981047	0.8069736	0.80686325	0.80647147
CHEMBL384575	0.8069736	0.80686325	0.80647147
CHEMBL3697935	0.80694073	0.8062469	0.8059883
CHEMBL3965901	0.80688655	0.80651426	0.8061765
CHEMBL4291202	0.80687255	0.80702204	0.80667484
CHEMBL294395	0.80685437	0.8063654	0.8063298
CHEMBL29714	0.80684674	0.8053335	0.8046826
CHEMBL547275	0.80676824	0.8063707	0.8061323
CHEMBL92902	0.80668306	0.8066586	0.8063635
CHEMBL69629	0.80662787	0.8060573	0.80583644
CHEMBL104896	0.80660313	0.80649585	0.80667543
CHEMBL2335870	0.8065666	0.8063378	0.8058412
CHEMBL3134612	0.8064729	0.8062185	0.80630344
CHEMBL3973156	0.8063188	0.80607694	0.8059652
CHEMBL3265012	0.8061981	0.80579305	0.805581
CHEMBL53555	0.8061949	0.80571723	0.80565995
CHEMBL3329404	0.80619216	0.8051288	0.8053223
CHEMBL3596856	0.8061813	0.80537844	0.8054812
CHEMBL1795832	0.8060726	0.8058788	0.8055101

CHEMBL4211854	0.8060558	0.80566025	0.80552506
CHEMBL4215341	0.8060558	0.80566025	0.80552506
CHEMBL3410029	0.80600345	0.8051749	0.8047411
CHEMBL2336805	0.8059802	0.80626875	0.8056076
CHEMBL30268	0.8059515	0.80495137	0.8044329
CHEMBL3290946	0.80591524	0.8055173	0.80513227
CHEMBL58936	0.80590326	0.8053901	0.80465925
CHEMBL3642594	0.80588156	0.8054812	0.80510885
CHEMBL308498	0.8058551	0.8055391	0.8051905
CHEMBL4172459	0.8058208	0.80561423	0.8053062
CHEMBL3099971	0.8058099	0.80577594	0.80502
CHEMBL3674283	0.8057622	0.805467	0.80516297
CHEMBL3221549	0.8057411	0.80524665	0.8051298
CHEMBL3633686	0.80571806	0.805465	0.8048584
CHEMBL3933100	0.8056438	0.8054999	0.80542016
CHEMBL179521	0.80556095	0.80515313	0.8047161
CHEMBL3623884	0.80549943	0.8051176	0.80476433
CHEMBL186905	0.80538356	0.8044885	0.80464983
CHEMBL467232	0.8053748	0.80398357	0.8034344
CHEMBL1088857	0.80528754	0.8050521	0.80492175
CHEMBL4163364	0.80528194	0.8050019	0.80521107
CHEMBL402524	0.8052744	0.80500245	0.8050923
CHEMBL343209	0.8052441	0.80479205	0.80489403
CHEMBL3679278	0.80514383	0.80494833	0.8040558
CHEMBL52845	0.8051427	0.80473304	0.8045627
CHEMBL442749	0.80510247	0.80467	0.80452317
CHEMBL3800352	0.8050211	0.80468184	0.8046268
CHEMBL97771	0.80491376	0.80485755	0.804876
CHEMBL3728263	0.80475837	0.8045475	0.8044435
CHEMBL1824292	0.80475664	0.80407965	0.8040655
CHEMBL3637864	0.804729	0.80442786	0.8042971
CHEMBL294034	0.8046666	0.8041847	0.80409586
CHEMBL4177008	0.8046645	0.80435467	0.80451417
CHEMBL120911	0.8046565	0.8049175	0.8051124
CHEMBL59652	0.8045924	0.80396545	0.80395687
CHEMBL2335889	0.8045921	0.8045293	0.80438143
CHEMBL2016600	0.8045677	0.8042816	0.80402684
CHEMBL3645272	0.80455	0.8041121	0.80386263
CHEMBL522644	0.8045192	0.8038717	0.80381477
CHEMBL3914525	0.80447304	0.80376816	0.8033301
CHEMBL588892	0.80446196	0.8041896	0.80366296
CHEMBL66868	0.8044503	0.8035933	0.8033663
CHEMBL419185	0.8042606	0.8031578	0.802746
CHEMBL373798	0.804235	0.8041055	0.8040027
CHEMBL3798342	0.80413496	0.8038373	0.80396676
CHEMBL3659924	0.8040384	0.80314946	0.8030278
CHEMBL4288482	0.8039744	0.8028494	0.8024813
CHEMBL249574	0.80396295	0.80424863	0.8037143
CHEMBL2436957	0.80394506	0.80379146	0.8035013
CHEMBL432903	0.80392843	0.8035552	0.803499

CHEMBL363085	0.80391514	0.8031773	0.80307496
CHEMBL238326	0.8038362	0.8036272	0.8032838
CHEMBL1551534	0.80380005	0.803486	0.8032185
CHEMBL261805	0.8037858	0.8034629	0.80308735
CHEMBL376307	0.80374044	0.8037464	0.80394316
CHEMBL586150	0.80373645	0.80347764	0.8029514
CHEMBL1506438	0.8037123	0.8034955	0.80333817
CHEMBL522941	0.8036943	0.8030037	0.8025988
CHEMBL182201	0.803691	0.8031553	0.8029323
CHEMBL4161581	0.80362654	0.8034216	0.803121
CHEMBL1914668	0.80359226	0.8031863	0.8032655
CHEMBL352877	0.80351037	0.8030537	0.80314463
CHEMBL369360	0.80351037	0.8030537	0.80314463
CHEMBL473553	0.8034626	0.80309075	0.80292964
CHEMBL479636	0.80340636	0.80294704	0.80223465
CHEMBL171125	0.80337256	0.8027598	0.8026853
CHEMBL3970316	0.80336726	0.80276835	0.80194193
CHEMBL4165320	0.8032181	0.8032611	0.8032297
CHEMBL1081166	0.803202	0.8027345	0.8026242
CHEMBL2029983	0.8031918	0.8023492	0.8022598
CHEMBL2417990	0.8031125	0.8029122	0.8025892
CHEMBL4224932	0.80309695	0.8020854	0.8016808
CHEMBL3939490	0.80302703	0.8026924	0.80263716
CHEMBL454868	0.8029647	0.802376	0.8023174
CHEMBL4168177	0.80296266	0.8025499	0.8025967
CHEMBL4168590	0.80295336	0.8024131	0.80209434
CHEMBL2179120	0.8029007	0.80280924	0.8027699
CHEMBL1172719	0.80288345	0.801807	0.801117
CHEMBL4099431	0.8028656	0.80241764	0.801938
CHEMBL3593287	0.80280197	0.8018369	0.8017732
CHEMBL3694147	0.80277085	0.8020732	0.80171204
CHEMBL2208026	0.80273974	0.80261487	0.8026463
CHEMBL1933080	0.8027326	0.8023098	0.8020568
CHEMBL3797998	0.80269694	0.8023739	0.8025011
CHEMBL264903	0.8026743	0.8015333	0.8012307
CHEMBL1240566	0.8026178	0.8021518	0.8013946
CHEMBL1208888	0.8026012	0.80244863	0.8024435
CHEMBL3697912	0.8025713	0.8020474	0.8016593
CHEMBL255955	0.8023528	0.8017215	0.80118674
CHEMBL2417873	0.80228966	0.8019323	0.8016665
CHEMBL1630654	0.8022026	0.80207413	0.80198133
CHEMBL4165375	0.8021945	0.80167246	0.8015835
CHEMBL468269	0.8021418	0.80086577	0.8004306
CHEMBL3730566	0.80213845	0.80179036	0.8014119
CHEMBL45582	0.80204237	0.80165863	0.8009669
CHEMBL492409	0.80196726	0.8009167	0.800679
CHEMBL1172718	0.8018575	0.800248	
CHEMBL256205	0.8018383	0.80140024	0.80111253
CHEMBL3951583	0.8018185	0.8021757	0.80140114
CHEMBL141852	0.8018068	0.8009192	0.8006482

CHEMBL1824288	0.8016732	0.8010973	0.8013562
CHEMBL1824294	0.8016732	0.8010973	0.8013562
CHEMBL1080394	0.80167	0.8018154	0.80185515
CHEMBL3740987	0.8016643	0.8013236	0.8011689
CHEMBL284326	0.8015616	0.8010246	0.80071074
CHEMBL3593672	0.80155975	0.800581	0.8005644
CHEMBL203599	0.8014803	0.80125177	0.8009764
CHEMBL271069	0.80145335	0.8014722	0.80202305
CHEMBL3654712	0.8014279	0.8016935	0.80140936
CHEMBL187144	0.80136836	0.8007328	0.8002889
CHEMBL1258242	0.8013563	0.80135524	0.8011347
CHEMBL355826	0.80134046	0.8010329	0.8007606
CHEMBL2335883	0.80128676	0.80160767	0.80134404
CHEMBL3633685	0.80128634	0.8013503	0.80144906
CHEMBL105364	0.8012626	0.8014528	0.801348
CHEMBL4208579	0.8012492	0.8003926	0.8000692
CHEMBL141453	0.801188	0.8007774	0.80112046
CHEMBL31639	0.8011066	0.8001887	
CHEMBL3221550	0.80110586	0.8007785	0.80063903
CHEMBL3694148	0.8010916	0.8001237	0.8001131
CHEMBL3694151	0.8010916	0.8001237	0.8001131
CHEMBL4165319	0.80108005	0.8007337	0.8005533
CHEMBL3646722	0.8008242	0.800267	0.80020857
CHEMBL1950890	0.80077326	0.8005563	0.8002497
CHEMBL3759884	0.80067027	0.80020314	
CHEMBL4164550	0.8006272	0.8000563	
CHEMBL3593659	0.8006241	0.8002442	0.8002745
CHEMBL1258267	0.80053127	0.80002964	
CHEMBL294711	0.8005011		
CHEMBL3732023	0.8004651	0.8001588	0.8000244
CHEMBL1314291	0.80044484	0.80061024	0.80019194
CHEMBL128432	0.8004039	0.8004699	0.800179
CHEMBL3658724	0.80035824	0.8003011	
CHEMBL435098	0.800249		
CHEMBL334169	0.80022514		
CHEMBL4173969	0.80020845		
CHEMBL3913441	0.80013347		
CHEMBL69358	0.80013144		
CHEMBL178953	0.8001279		
CHEMBL3680508	0.8000667	0.8001535	
CHEMBL3728522	0.8000542		
